# Expression of MicroRNAs in Sepsis-Related Organ Dysfunction: A Systematic Review

**DOI:** 10.3390/ijms23169354

**Published:** 2022-08-19

**Authors:** Aniello Maiese, Andrea Scatena, Andrea Costantino, Enrica Chiti, Carla Occhipinti, Raffaele La Russa, Marco Di Paolo, Emanuela Turillazzi, Paola Frati, Vittorio Fineschi

**Affiliations:** 1 Department of Surgical Pathology, Medical, Molecular and Critical Area, Institute of Legal Medicine, University of Pisa, 56126 Pisa, Italy; 2 Department of Clinical and Experimental Medicine, University of Foggia, 71122 Foggia, Italy; 3 Department of Anatomical, Histological, Forensic and Orthopedic Sciences, Sapienza University of Rome, Viale Regina Elena 336, 00161 Rome, Italy

**Keywords:** sepsis, microRNA, miRNA, multiple organ dysfunction, brain, heart, lungs, liver, kidney, blood

## Abstract

Sepsis is a critical condition characterized by increased levels of pro-inflammatory cytokines and proliferating cells such as neutrophils and macrophages in response to microbial pathogens. Such processes lead to an abnormal inflammatory response and multi-organ failure. MicroRNAs (miRNA) are single-stranded non-coding RNAs with the function of gene regulation. This means that miRNAs are involved in multiple intracellular pathways and thus contribute to or inhibit inflammation. As a result, their variable expression in different tissues and organs may play a key role in regulating the pathophysiological events of sepsis. Thanks to this property, miRNAs may serve as potential diagnostic and prognostic biomarkers in such life-threatening events. In this narrative review, we collect the results of recent studies on the expression of miRNAs in heart, blood, lung, liver, brain, and kidney during sepsis and the molecular processes in which they are involved. In reviewing the literature, we find at least 122 miRNAs and signaling pathways involved in sepsis-related organ dysfunction. This may help clinicians to detect, prevent, and treat sepsis-related organ failures early, although further studies are needed to deepen the knowledge of their potential contribution.

## 1. Introduction

Sepsis is defined as a potentially fatal systemic dysfunction caused by a dysregulated host response to infection. The consequences of this process can lead to septic shock and the dysfunction or failure of vital organs. Clinically, according to the most recent consensus conference (Sepsis-3), an increase of at least two points from baseline on the SOFA (Sequential Organ Failure Assessment) scale is required to diagnose sepsis [1]. Critically ill patients, especially those with significant comorbidities, are prone to multi-organ failure (MOF) [2]. In hospitals, especially in countries with higher life expectancy, sepsis is a very common condition [3]. However, due to this disease’s high morbidity and mortality, sepsis imposes very high costs on the healthcare systems of most developed countries [4]. For this reason, sepsis is currently one of the most important health problems on which biomedical research should focus to find new early diagnostic methods and new selective therapies. Although many efforts have been made so far to study this pathological process, there are still many questions to be answered about the pathogenesis and pathophysiology by which damage to vital organs occurs [5]. The degree of dysfunction of the affected organs can range from mild impairment to irreversible failure and depends largely on the delicate balance between pro-inflammatory and anti-inflammatory factors [6]. Indeed, the interaction between microbial components, such as bacterial LPS lipid A, and monocytes and macrophages releases pro-inflammatory immunomodulators, such as TNF-α and IFN-γ, into the bloodstream within a few hours. However, these immunomodulators trigger a self-stimulatory process that activates the release of other pro-inflammatory mediators such as interleukins (IL -1, IL -2, IL -6, IL -8) and PAF, or suppresses the release of anti-inflammatory mediators (IL -10), thereby increasing the concentration of circulating cytokines. This uncontrolled inflammatory cascade leads to the constant activation of polymorphonuclear leukocytes, macrophages, and lymphocytes and generates a self-destructive immunological dissonance [7]. The organs most affected by this phenomenon are the circulatory system, including the vessels and the heart, the lungs, the central nervous system, the liver, and the kidney, which can be irreversibly damaged [8]. Given the recent findings on the role of non-coding RNA (ncRNA) in regulating biological processes (cell proliferation, differentiation, apoptosis and cycle regulation), more and more studies are focusing on the use of these molecules as diagnostic markers or for therapeutic purposes [9,10]. One of the most studied classes of ncRNAs is that of microRNAs (miRNA or miR). miRNAs are small molecules consisting of 19–25 ribonucleotides that are not translated into proteins but have the ability to regulate the expression of specific target genes in the post-transcriptional phase [11]. Given their role in epigenetic regulation and their tissue, intracellular, and extracellular ubiquity, miRNAs have the potential to be used not only as serum markers during sepsis but also as specific markers to assess the involvement of individual organs in this pathological process [12,13]. By understanding which biological processes are regulated by specific miRNAs during sepsis, new tailored and ultra-selective therapies can be developed. For this reason, a review of the currently available data on miRNAs involved in the major target organs (heart, lung, liver, kidney and blood) during sepsis has been carried out.

## 2. Materials and Methods

### 2.1. Eligibility Criteria

The present systematic review was carried out according to the Preferred Reporting Items for Systematic Review (PRISMA) standards [14]. We used an evidence-based model for framing a PICO.

### 2.2. Search Criteria and Critical Appraisal

A systematic literature search and a critical appraisal of the collected studies were conducted. An electronic search of PubMed, Science Direct Scopus, and the Excerpta Medica Database (EMBASE) from the inception of these databases to 10 February 2021 was performed. The search terms were: “miRNA expression in brain tissue during sepsis”; “miRNAs in brain tissue sepsis”; “Sepsis induced lung injury”; “Sepsis induced myocardial injury”; “miRNAs expression in liver tissue during sepsis”; “liver miRNAs during sepsis”; “sepsis and miRNA expression in liver”; “miRNA expression during sepsis-induced Acute Kidney Injury”; “MiRnas sepsis markers in blood”; “circulation sepsis marker” in the title, abstract and keywords. The bibliographies of all identified documents were reviewed and cross-checked for other relevant literature. A methodological assessment of each study was conducted according to PRISMA standards, including the assessment of bias. Data collection included study selection and data extraction. Three researchers (A.S., C.O., E.C.) independently reviewed those documents whose title or abstract seemed relevant and selected those that analysed miRNAs. The question of the suitability of miRNAs was resolved by consensus among the researchers. Unpublished or grey literature was not searched. Data extraction was performed by three investigators (E.T., A.M., A.C.) and reviewed by two other researchers (V.F., P.F.). Only English-language papers or abstracts were included in the search.

## 3. Results

### 3.1. Search Results and Included Studies

An appraisal based on titles and abstracts and a hand search of reference lists were carried out. The reference lists of all located articles were reviewed to detect still unidentified literature. This search identified 5192 articles which were then screened based on their abstract to identify their relevance in respect to the following:

Estimated diagnosis process;

Clinical features analyzed;

Circumstantial data evaluation;

Study design.

**Figure 1:**
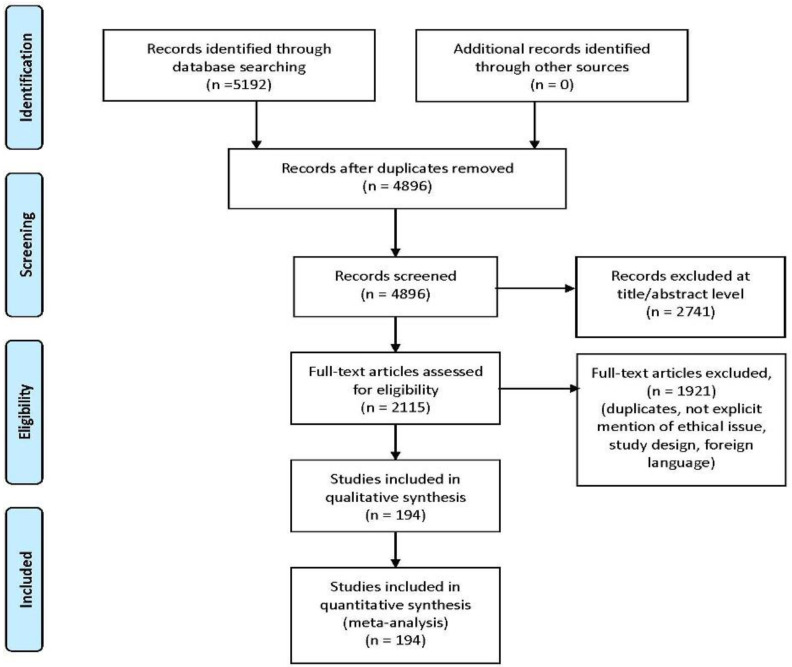
Preferred Reporting Items for Systematic Review (PRISMA) flow chart—search strategy.

The methodology of our search strategy is represented in Figure 1.

Preferred Reporting Items for Systematic Review (PRISMA) flow chart—search strategy. Study designs comprised retrospective and prospective studies, original articles, and reviews. An appraisal based on titles and abstracts and a hand search of reference lists were carried out. The reference lists of all located articles were reviewed to detect still unidentified literature. A total of 60 studies fulfilled the inclusion criteria.

Study designs comprised retrospective and prospective studies, original articles, and reviews. An appraisal based on titles and abstracts and a hand search of reference lists were carried out. The reference lists of all located articles were reviewed to detect still unidentified literature. A total of 194 studies fulfilled the inclusion criteria.

### 3.2. Risk of Bias

This systematic review has a number of strengths, including the amount and breadth of the studies, which span the globe; the hand search and scan of reference lists for the identification of all relevant studies; and a flowchart that describes in detail the study selection process. It must be noted that this review includes studies that were published in a time frame of 5 years; thus, despite our efforts to fairly evaluate the existing literature, study results should be interpreted taking into account that the accuracy of clinical procedures, where reported, has changed over the years.

### 3.3. Expression of miRNAs in Sepsis-Related Organ Dysfunction

#### 3.3.1. miRNA Expression in Sepsis-Related Brain Dysfunction

Sepsis is a multi-organ disease that can affect the brain via several processes, including neuroinflammation and ischemia, oxidative stress, microglial activation and microcirculatory dysfunction, which damage neurons interfering with normal brain function and cognitive impairment [15]. MiRNAs are biomolecules with an increasingly important role in many biological functions and have been postulated to contribute not only to normal brain function but also to neuropathological conditions [16]. Many miRNAs are evaluated as potential biomarkers in sepsis, because altered miRNA expression is described in septic conditions. For example, circulating miR-494-3p levels are decreased in sepsis patients compared to controls, while TLR6 is simultaneously upregulated. Similar conditions are reported from LPS-induced RAW 624.7 cells, with low miR-494-3p levels and high TNF-α and TLR6 levels. miR-494-3p seems to attenuate the sepsis-associated inflammatory response by controlling TLR6 expression [17]. In a recent study by Tong et al., high levels of miR-146b-5p were associated with suppressed KLF4 expression during intestinal injury in a rat model of sepsis [18].

Unfortunately, only few recent studies have analyzed sepsis and the altered expression of miRNAs in brain tissue.

Dong et al. analysed the protective role of miR-181b in aged rats with sepsis-induced hippocampal injury, showing significantly decreased miR-181b expression in septic rats compared to controls. High miR-181b levels were associated with lower hippocampal inflammation, the inhibition of the NF-κB pathway, and downregulation of the inflammatory cytokines IL-1β and TNF-α [19]. miR-181b upregulation was found instead in the cerebral cortex (and serum) of septic rats by Chen et al. [20].

Yu et al. described miR-200a-3p overexpression in human brain microvascular endothelial cells compared to controls, along with the increased expression of ROS, IL-1β and IL 18. In contrast, levels of Keap1, Nrf2 and HO1 were reduced. miR-200a-3p overexpression seems to promote the inflammatory response through NLRP3 induced by ROS [21].

Visitchanakun et al. instead analysed miR-370-3p expression in mice and human patients with sepsis-associated encephalopathy (SAE), identifying high miR-370-3p levels in mice brain and plasma. miR-370-3p overexpression increased TNF-α-related brain apoptosis in SAE mice. SAE patients showed high miR-370-3p plasma levels compared to controls [22].

Kim et al. developed a transgenic mouse model of systemic inflammation and found miR-147 downregulation in brain compared to other organs (i.e., lung, kidney and stomach) and to controls [23].

Nong et al. reported miR-126 downregulation in experimental septic rats compared to controls. Increased miR-126 brain expression was related to low sepsis-induced blood–brain barrier damage (inflammation and excessive oxidative stress), due to the inhibition of the NF-κB pathway [24].

Rani et al. instead analyzed the hippocampus of septic mice (at day 1 and day 4 after sepsis), evaluating the influence of age and sex. A few miRNAs, generally associated with neuroprotection against inflammation, showed similar changes with age and sex: miR-190a-3p, let-7a-1-3p, miR-3085-3p were upregulated in young and older females at day 1, while miR-383-5p was downregulated in young males and females at day 4 [25].

Zou et al. analyzed miR-146a knock-out mice, describing a significant increase in neutrophils and monocytes in the brains of septic WT mice compared to the knock-out group [26]. Cultured microglia and astrocytes treated with miR-146a mimic showed marked chemokine CXCL2 production in microglia and lower CXCL2 production in astrocytes. These results suggest that miR-146a downregulation could attenuate the inflammatory response during sepsis in brain tissue.

miRNA expression in sepsis-related brain dysfunction are summarized in Table 1.

#### 3.3.2. miRNA Expression in Sepsis-Related Heart Dysfunction

One of the most affected organs in sepsis is the heart. There are several mechanisms potentially responsible for sepsis-induced cardiomyopathy, such as the downregulation of adrenergic pathways, decreased calcium response [27,28], disturbances in microcirculatory endothelial function [29,30,31], mithocondrial dysfunction [32,33,34,35], and the release of nitric oxide and reactive oxygen species [36]. All these phenomena seem to be interdependent and in turn depend on the main cause of sepsis: inflammation [37,38]. Indeed, PAMPs and DAMPs are triggers for the activation of numerous intracellular signalling pathways through the inscription of Toll-like receptors, including nuclear factor-κB, thus stimulating cell proliferation. Moreover, the activation of Toll-like receptors in monocytes and macrophages promote the production of cytokines that directly affect cardiac muscle contractility [39]. How these changes in circulating immune cells and inflammatory mediators relate to septic cardiomyopathy is poorly understood. However, an increasing number of studies point to the role of miRNA as regulators of such processes. This leads us to consider them not only as biomarkers but also as potential therapeutic targets for the treatment of sepsis-induced cardiomyopathy, in particular, and sepsis in general [40,41,42,43,44,45,46,47,48,49]. Cardiac involvement and impairment with subsequent heart failure is one of the leading causes of death in septic patients, and this also applies to long-term cardiovascular effects. Persistent inflammatory activation, indeed, is notably linked to the development and progression of atherosclerosis, as well as to an increased risk of stroke and myocardial infarction [50,51]. Chronic inflammation may also predispose the onset of atrial fibrillation [52,53].

These reasons highlight the importance of the early detection of sepsis-induced cardiomyopathy, and the role of miRNA may be key to this end.

Wang et al. investigated the miRNA expression profiles in the myocardial tissues of mice treated with cecal ligation and puncture (CLP) [54]. They found that miR-223 was downregulated after severe CLP surgery, leading to an increase in the levels of Sema3A and Stat3/IL6. This response then triggers an inflammatory reaction and depression of the myocardium. The decrease in serum levels of miR-223 was also found in patients who died of sepsis, supporting these findings [55,56]. Molecular studies have shown that miR-223 suppresses semaphorin-3A (Sema3A), an inducer of cytokine storm, signal transducer and activator of transcription 3 (STAT -3), and interleukin (IL)-6, and therefore its downregulation may cause the upregulation of these pro-inflammatory mediators.

A multi-omics study has also shown that MiR-223 plays an important role in the control of the NF-κB system and is involved in the regulation of inflammation (e.g., in monocyte differentiation) [57].

Xue et al. showed that miR-27a expression was increased in the myocardium of mice exposed to lipopolysaccharide (LPS), determining a reduction in cardiac function [58]. They also performed in vitro experiments to understand the mechanism of action: miR-27a was found to be involved in regulating the expression of nuclear factor (erythroid-derived)-like 2 (Nrf2). Nrf2 is a transcription factor that modulates the expression of antioxidant enzymes [59]. Furthermore, in 2015, Gao et al. demonstrated that an increased expression of miR-146a attenuated myocardial dysfunction in polymicrobial sepsis by inhibiting NF-κB activation [60]. In 2018, An et al. confirmed the attenuation of miR-146a-induced inflammation in an in vitro experiment [61]. Specifically, they found that the overexpression of miR-146a significantly increased the expression of ErbB4, decreased the expression of TRAF6, IRAK1, caspase 3 and even the activation of NF-κB, and also increased the Bcl-2/Bax ratio, suggesting its role in the inhibition of inflammation and apoptosis.

Recently, Xie et al. confirmed these results and indicated that miR-146a could affect the TLR -4/NF-κB signaling pathway [62]. Wang et al. demonstrated that miR-146b also plays a protective role against sepsis-induced cardiomyopathy [63]. They suggested that it acts by inhibiting Notch1, which is normally involved in cardiac development and inflammatory processes.

In another work, Ma et al. demonstrated a similar protective effect played by miRNAs. They found that such an effect was achieved through the increased expression of miR-125b [64]. Moreover, cardiac function improved when a miR-125b mimic was transfected. They also investigated the role of the long non-coding RNA (lncRNA), MALAT1. They concluded that there is a correlation between MALAT1, miR-125b decrease, and the p38 MAPK/NF-κB signaling pathway, leading to the possibility that MALAT1 may increase myocardial inflammation, contrasting the protective role of miR-125b. MALAT1 was even the subject of Wei and Liu’s work [65]. They found that miR-150-5p is inhibited by MALAT1 and, therefore, they play opposing roles in sepsis-induced myocardial inflammation. Indeed, whereas the overexpression of miR-150-5p is protective, the overexpression of MALAT1 exacerbates cardiac inflammation. It also appears that miR-150-5p regulates the NF-κB signaling pathway. A recent study confirmed the protective effect of miR150-5p on myocardial cells in sepsis [66].

In two different studies, Wang et al. investigated the changes in the expression of miR-21-3p and miR-155 in the heart tissue of mice exposed to LPS [67,68]. Both miR-21-3p and miR-155 were upregulated in cardiac tissues after the intraperitoneal injection of LPS. Indeed, the administration of their relative antagomiRNA (antagomiR) prior to LPS exposure ameliorated cardiac dysfunction, and conversely, the prior administration of agomiRNA (agomiR) worsened cardiac dysfunction. Notably, the development of an agomiR for miR-155 improved cardiac function and suppressed apoptosis by targeting Pea15a. To evaluate the clinical relevance of their findings, they also measured miR-21-3p in the blood of septic patients with cardiac involvement and found that this level was higher than in septic patients without cardiac dysfunction. Diao and Sun performed a similar work [69]. They investigated the expression of miR-124a in the myocardium of mice with LPS-induced sepsis and its change after the administration of antagomiR and agomiR. MiR-124a was downregulated in septic mice, whereas its inhibition and stimulation led to the deterioration and improvement of cardiac function, respectively.

A very interesting study was performed by Zheng et al. [70]. First, they measured the level of miR-135a in the serum of patients with sepsis-induced cardiac depression and found a correlation between the level of miR-135a and the severity of myocardial dysfunction. Then, they performed CLP surgery in miR-135a-transfected mice. Myocardial inflammation was more severe in the transfected mice than in the non-transfected mice, while cardiac function was decreased. They also suggested that the pro-inflammatory effect of miR-135a might be mediated by the activation of the p38 MAPK/NF-κB pathway.

Zhou et al. found that the injection of a miR-155 mimic exerted a protective effect on cardiac dysfunction after CLP surgery in mice and that the prior transfection of miR-155 reduced the infiltration of inflammatory cells into the myocardium [71]. The authors also suggested that miR-155 inhibited the expression of β-arrestin 2 (Arrb2), a protein involved in the regulation of the immune system. Other authors have pointed out the upregulation of miR-214 during sepsis in mice [72]. In their study, myocardial inflammation, apoptosis, and dysfunction were decreased when miR-214 expression was increased by its precursor, and conversely, they were worsened by its inhibitor. In another study, miR-214-3p was shown to inhibit autophagy via the PTEN/AKT/mTOR pathway [73].

A study on the expression of miR-874 was performed by Fang et al. [74]. They found that miR-874 was upregulated in the serum of sepsis patients, in LPS-induced sepsis mice, and even in the myocardial cells of septic mice. They also found a negative correlation between miR-874 and the lncRNA H19 and aquaporin 1 (AQP1), suggesting that they perform opposite roles. In particular, it seems that miR-874 worsens inflammation and myocardial function. Previous studies have shown that AQP1 is involved in other pathophysiological mechanisms such as tumor development, inflammatory cytokine release (via the NF-κB pathway), and polycystic kidney disease (via the Wnt pathway) [75,76,77].

In the work of Tang et al., MiR-93-3p was downregulated in LPS-treated cells [78]. It appeared to be involved in the regulation of Toll-like receptor 4 (TRL4) translation. The overexpression of miR-93-3p suppressed apoptosis and cytokine production, suggesting a protective role against septic-induced cardiac injury. The function of another miRNA was investigated by Yao et al. [79]. They found that miR-25, which is decreased during sepsis, exerts a protective effect against apoptosis in LPS-induced cell injury when overexpressed, thus reducing myocardial injury. They suggested that miR-25 affects the TLR4/NF-κB signaling pathway and directly targets the phosphatase and tensin homolog (PTEN).

Wu et al. found that miR-494-3p also targets PTEN [80]. According to their results, miR-494-3p is downregulated in the blood of septic patients, and its decrease correlates with cardiac dysfunction. On the other hand, the upregulation of miR-494-3p protects rat cardiomyocytes from apoptosis. Zhang et al. found that MiR-23b was increased in the myocardium of CLP mice [81]. Its inhibition not only reduced myocardial dysfunction but also decreased cardiac remodeling. The authors noted that the target gene of miR-23b is 5’TG3’-interacting factor 1 (TGIF1), which in turn inhibits transforming growth factor β1 (TGF- β1), which is known to be involved in fibrogenesis [82,83]. However, it should be noted that Cao et al. reported opposite results [84]. In their study, miR-23b appeared to attenuate sepsis-induced cardiac dysfunction. They upregulated miR-23b in cardiomyocytes both in vivo and in vitro and then measured cardiac function and the secretion of inflammatory cytokines. These were increased and decreased, respectively. They also found a decreased expression of adhesion molecules, the activation of the NF-κB pathway, and caspase-3 activity. Unfortunately, Cao et al. did not investigate the reasons for this difference. Perhaps it is reasonable to hypothesize different pathways for the two strands of the same pri-miRNA.

Guo et al. found that MiR-495 was downregulated in blood samples from septic patients [85]. Moreover, the decrease was even more pronounced in patients who developed septic shock. The authors, who created a rat sepsis model by CLP modeling, found that there was a decrease in MiR-495 in the myocardium and serum of septic rats. Cardiac function was also impaired. The injection of agomiR-495 reduced inflammation and reversed myocardial dysfunction.

Zhu et al. [86] investigated the role of miR-98 in cardiac dysfunction caused by sepsis. They discovered that the expression of miR-98 was downregulated in the myocardium of CLP mice. In contrast, mice that received an injection of agomiR-98 showed improved cardiac function, less myocardial damage and apoptosis (by inhibiting cleaved caspase-3 and the Bax protein), and a different pattern of cytokines. Specifically, tumor necrosis factor α (TNF- α) and interleukin-6 (IL -6) were increased, whereas IL-10 was decreased. Another study showed that miR-208a-5p was increased in the myocardium during sepsis and its inhibition could reduce the myocardial damage caused by inflammation, probably by affecting the NF-κB/HIF-1α pathway [87].

Sun et al. found a positive correlation between miR-328 serum levels and sepsis in human patients [88]. Moreover, miR-328 appeared to correlate with cardiac dysfunction in the CLP rat model, while the injection of miR-328 antagomiR appeared to reduce inflammation and improve impairment. In 2019, Zhang et al. identified 78 miRNAs that were differentially expressed according to the stage of the septic process in rat heart [89]. They then constructed a complex network to show the relationship between miRNAs and circular RNAs (circRNAs), another type of non-coding RNA involved in the control of biological pathways via the inhibition of miRNAs [90].

In a recent study by Zhu et al., MiR-29a expression appeared to be increased in sepsis [91]. They investigated the role of an lncRNA, CRNDE, in sepsis-induced myocardial injury. CRNDE decreased apoptosis, inflammation, and oxidative stress in cardiomyocytes treated with LPS by inhibiting miR-29a. Contrasting results were provided by Song et al. [92]. In their study, miR-29a appeared to be downregulated by LPS and to play a positive role in preventing and attenuating cardiac injury. The lncRNA CYTOR was investigated by Chen et al. [93]. CYTOR regulates the expression of miR-24, which inhibits the translation of X chromosome-linked apoptosis inhibitor (XIAP). As a result of this pathway, the downregulation of CYTOR or the upregulation of miR-24 appeared to exacerbate sepsis-induced myocardial injury via the activation of apoptosis. Sun et al. demonstrated the role of the lncRNA KCNQ1OT1 in regulating miR-192-5p in myocardial injury induced by sepsis [94]. KCNQ1OT1 downregulated miR-192-5p, which in turn inhibited the translation of XIAP, improved myocardial cell viability, and counteracted apoptosis.

Nuclear Enriched Abundant Transcript 1 (NEAT1) is another lncRNA that was found to be implicated in sepsis-induced myocardial injury in a recent study [95]. NEAT1 exerts negative control on miR-144-3p. NEAT1 was found to be increased in cardiac myocytes when LPS was administered, whereas miR-144-3p was decreased. MiR-144-3p appeared to be involved in apoptosis and inflammation in cardiac myocytes via the NF-κB pathway. Xing et al. investigated the role of lncRNA myocardial infarction-associated transcript (MIAT) in sepsis-induced myocardial injury [96]. MIAT appeared to be a downregulator of miR-330-5p. In this study, miR-330-5p attenuated myocardial oxidative stress and inflammatory response thanks to its target protein, tumor necrosis factor receptor-associated factor 6 (TRAF6), which is involved in NF-κB signaling. MiR-330-5p is downregulated in septic myocardial cells.

In another work, the interaction between the lncRNA component of mitochondrial RNA processing (RMRP) and miR-1-5p was investigated [97]. In this work, the protective role of RMRP against sepsis-induced myocardial and mitochondrial damage was described. RMRP inhibits miR-1-5p, which in turn targets the heat shock protein HSPA4 (formerly known as hsp70). Such pathway results in reduced myocardial inflammation and injury.

Another study by Li and coworkers [98] showed that LPS treatment decreased the level of miR-29b-3p in mouse hearts treated with LPS. When this miRNA expression was induced, cardiac injury decreased and cardiac function improved. The molecular mechanism mediating this beneficial effect was related to the direct targeting of the transcription factor FOXO3A by miR-29b-3p.

Recent studies have identified additional miRNAs that may be involved in sepsis-induced cardiomyopathy. The group of Ye et al. focused on the role of miR-101-3p [99]. In this study, both in vivo and in vitro models of myocardial damage were established. It was found that miR-101-3p levels were significantly increased in the serum of patients with sepsis-induced cardiomyopathy and that there was also a positive correlation between the upregulation of miR-101-3p and the increasing levels of pro-inflammatory cytokines (IL -1, IL -6 and TNF, in particular). Similar results were obtained by creating a rat model for sepsis-induced cardiomyopathy. To evaluate the effects of miR-101-3p, rats were treated with miR-101-3p inhibitors. As a result, the expression of miR-101-3p was significantly downregulated, leading to a reduction in cardiac cell injury, inflammatory cell infiltration, and apoptosis. The expression levels of IL -1β, IL -6, and TNF-α were also significantly downregulated. The study also found that miR-101-3p acts by targeting the inhibition of DUSP1, a phosphatase capable of suppressing the MAPK signaling pathways p38 and NF-κB.

A study conducted by Yjuan et al. discovered the involvement of miR-106-5p. Specifically, that the role of miR-106-5p is embedded in the matrine/PTENP1/miR-106-5p axis. Matrine is a bioactive compound derived from Sophora flavescens, Sophora alopecuroides, and Sopho-raonkinensis, and its administration has been associated with important cardioprotective effects. This result is achieved by the overexpression of miR-106-5p and the downregulation of the PTENP1 signaling pathway. The latter is a long non-coding RNA associated with the AKT signaling pathway, which plays an important role in regulating cellular behaviors such as cell growth, survival, and migration.

Such results were obtained both in vivo and in vitro [100].

Bingyu et al. even found that the serum level of miR-29c-3p was significantly increased in sepsis patients [101]. Moreover, the serum miR-29c-3p level was significantly higher in sepsis patients with cardiac dysfunction than in sepsis patients without cardiac dysfunction. This suggests that abnormal miR-29c-3p level is not only involved in the occurrence of sepsis but also associated with the condition and severity of sepsis. They also investigated the diagnostic significance of miR-29c-3p in sepsis and found that it has a sensitivity of 80.2% and a specificity of 81.1%.

The role of miR-29c-3p was further elucidated using the results from the rat CLP model. MiR-29c-3p level in CLP rats was inhibited after the intravenous administration of miR-29c-3p antagomir. As a result, cardiac function was shown to improve significantly, and the inflammatory response triggered by sepsis was downregulated.

Finally, Yang and Wen focused on the role of miR-499a-5p [102] and found that its level was significantly decreased in patients with sepsis-induced cardiomyopathy and that even the patients who survived had higher levels of the aforementioned miRNA compared with those who died from sepsis. The diagnostic value of miR-499a-5p was investigated and found to have a sensitivity of 67% and a specificity of 80%.

They also investigated the effect of miR-499a-5p in vitro by transfecting a miR-499a-5p mimic into AC16 cells. The result was increased cell viability in AC16 cells treated with LPS and miR-499a-5p mimic. Consequently, after the overexpression of miR-499a-5p, H9c2 cell apoptosis and LDH activity were decreased, which promoted the ability of miR-499a-5p to alleviate H/R-induced cardiomyocyte injury. In this study, miR-499a-5p was able to regulate sepsis-induced cardiomyopathy by targeting EIF4E, a translation initiation factor involved in the mRNA–ribosome binding step of eukaryotic protein synthesis.

miRNA expression in sepsis-related heart dysfunction are summarized in Table 2.

#### 3.3.3. miRNA Expression in Blood during Sepsis

As sepsis is a condition that needs to be controlled with antibiotics as soon as possible, it is crucial to recognize the onset of sepsis so that the right treatment can be administered to the patient in time.

The population group most affected by the vagueness of the diagnosis is the elderly, especially those with oncological issues or that are immunocompromised. There are often confounding factors such as chronic inflammation and changes in body temperature. To detect sepsis at an early stage, blood culture tests are nowadays performed. These analyses are characterized by inherent limitations, as pathogens in the bloodstream (bacteremia) detected via blood cultures are considered definitive evidence of sepsis in patients with compatible clinical criteria. However, although this approach has a very low detection limit (a few pathogens in a sample of 30–50 mL of blood), sepsis is correctly diagnosed in only 20–40% of cases [103].

From these prerequisites, it is easy to deduce that the diagnostic specificity is very high, while the sensitivity is very low. Furthermore, measuring changes in the host’s response to infection could be a faster alternative to blood culture for diagnosing sepsis. Indeed, many diagnostic algorithms include measurements of serum biomarkers, which can be used in addition to vital signs and epidemiological data to predict sepsis cases [1]. However, biomarker measurements for sepsis diagnosis can also be misleading. Firstly, biomarker levels fluctuate with the progression of sepsis and these time-dependent concentration fluctuations are different for each biomarker [104]. This means that biomarker measurements must be rapid to accurately reflect the patient’s condition and that more than one biomarker should be measured to detect sepsis, regardless of disease stage. On the other hand, most biomarkers used for sepsis diagnosis are inflammatory biomarkers whose levels can be altered by other conditions such as trauma, surgery, cancer or nephropathies. Therefore, biomarker values should always be interpreted in the context of a personalized assessment that takes into account all variables of the individual patient. The above problems could be significantly mitigated by the use of biosensors with micro- and nanostructured components for the frequent and rapid determination of multiple sepsis biomarkers [105,106].

Because of these characteristics, especially the fact that these biomarkers could be essential for certain types of septic patients, this topic is receiving a lot of attention in the international research community.

Despite this potential, there are currently no applications in clinical routine, despite the great potential for the prognostic stratification of patients.

Several miRNAs are affected in the course of sepsis. There are numerous research groups that have demonstrated an altered transcriptional expression of these small non-coding RNAs. For example, plasma levels of miR-494-3p were observed to decrease in patients with sepsis compared to healthy controls; this process correlates with increased levels of TLR6.

Furthermore, the expression of miR-494-3p decreased in LPS-induced RAW 264.7 cells, in parallel with the upregulation of TLR6 and TNF-α. This is because the increase in miR-494-3p in RAW 264.7 cells decreases TNF-α levels and suppresses the translocation process of NF-κB p65 into the nucleus. Thus, TLR6 has been shown to be the target of miR-494-3p. In summary, miR-494-3p attenuates the inflammatory response associated with sepsis by affecting TLR6 expression [17].

One of the many mechanisms involved in the pathogenesis of sepsis is miR-218, which may be involved in reducing the inflammatory response by decreasing the expression of VOPP1 via the JAK /STAT axis [107].

In addition, miR-122 plays a crucial role in the septic process and has a higher diagnostic value than CRP and leukocyte count. In particular, it has been shown to be useful in the differential diagnosis between sepsis and pathogenic dissemination from a wound. It has also been shown to be a prognostic marker for sepsis, albeit with low specificity and sensitivity [108].

In experiments on mouse models, it was observed that reducing miR-208a-5p levels while increasing SOCS2 leads to a reduction in LDH and MDA and an increase in SOD activity. Furthermore, this reduction in miR-208a-5p correlates with low levels of TNF-α, IL -6, NF-κB p65 and HIF-1a. MiR-208a-5p is also associated with the regulation of mitochondrial swelling. Its silencing leads to a reduction in this process. In summary, the role of this miRNA is to modify cardiac apoptosis in animal models via the NF-κB/HIF-1a axis, leading to a reduction in myocardial damage following septic stimulation [87].

Studies have shown that LPS-induced pyroptosis via the ACS pyroptosome and septic shock are inhibited by the downregulation of miR-21 [109]. On the other hand, there is evidence that the upregulation of miR-21 reduces inflammation and apoptosis [110]. Further confirmation is provided by studies showing that exosomes derived from bMSCs are able to reduce the clinical manifestations of sepsis and prolong survival, at least in animal models, by upregulating miR-21 [111].

During sepsis, it has been observed in both humans and animals that miR-328 is one of the miRNAs that experiences dysregulation. By determining this ribonucleotide chain, it is possible to diagnose a septic state so that it can be used as a diagnostic biomarker. The role of miR-328, especially when downregulated, may be to modify the inflammatory response of the myocardium during sepsis, which has crucial implications for cardiac dysfunction [88].

Finally, concentrations of miR-452 in serum and urine can be used as markers of sepsis. Indeed, high levels of this miRNA have been found in patients with acute kidney injury during sepsis, especially compared to septic patients without kidney injury [112].

In summary, miRNAs may play a role as prognostic and diagnostic markers in the early detection of septic processes or in the differentiation between sepsis and other inflammatory diseases. In particular, their use can be very useful in identifying organism damage in the different districts affected by sepsis. Among these, miRNAs that can be identified in blood have the greatest potential for use as biomarkers because they are easy to collect and test. miRNA expression in blood during sepsis are summarized in Table 3.

#### 3.3.4. miRNA Expression in Sepsis-Related Lung Dysfunction

Another organ often involved in sepsis is the lung. Indeed, acute lung injury (ALI) is one of the most common complications of sepsis, and one third to one half of sepsis will worsen to ALI.

ALI is actually the result of lung cell apoptosis triggered by the upregulation of inflammatory and apoptotic pathways. This leads to the disruption of alveolar epithelial cells, increases in epithelial permeability, low lung compliance, noncardiogenic pulmonary edema, and hypoxemia, thus erupting in acute respiratory distress syndrome (ARDS) [176,177,178].

The development of such syndrome is responsible for the high rates of morbidity and mortality of sepsis.

However, little is known about the pathophysiology of sepsis-induced ALI and ARDS. Consequently, the only available treatment for such complications consists of antibiotics and supportive measures, which exert very little impact on the high mortality of these conditions.

On the other hand, current target therapies such as antioxidants [179,180,181,182], prostaglandin E1 [183,184], neutrophil elastase inhibitors [185], activated protein C [186], statins [187,188], and keratinocyte growth factor [189] have not demonstrated effective benefits.

An increasing number of studies are highlighting the more and more important involvement of miRNAs in the development of such syndromes, thus allowing us to clarify the molecular pathways of sepsis-induced ALI and ARDS in order to better diagnose and treat them.

One of the most examined miRNAs is miR-155. It plays a relevant role in inflammation as it is highly expressed in lymphoid cells and mildly expressed in human lungs [190]. Indeed, miR155 has been demonstrated to be increased in several sepsis-induced ARDS models, including CLP ones [191]. Moreover, the collection of serum exosomes charged with miR-155 injected in previously healthy mice from mice with acute lung injury induced lung inflammation [192].

Several studies have indicated that these effects are mediated through the inhibition of suppressor of cytokine signaling-1 (Socs-1) [192,193], a negative regulator of inflammatory cytokines such as interferon (IFN) α/β/γ, IL-12/23, IL-4/13, and the IL-2 family cytokines [194].

A study from Hsiao-Fen Li also found that miR-155 induces inflammation through the inhibition of IRF2BP2, which in turn inhibits NFAT1, a transcription factor involved in cytokine production [195].

Han et al. evaluated the prognostic value of miR-155, finding that it could also be used for predicting the mortality and treatment outcome of sepsis-induced lung injury [196]. They also valued the prognostic value of miR-146, another miRNA which has been found to be involved in ALI and ARDS in numerous studies as well. All studies about miR-146 have demonstrated that it plays a protective role in inflammation and in experimental models of ARDS.

Indeed, the overexpression of miR-146 is associated with a reduction in inflammation and the attenuation of lung injury in response to acid aspiration and intratracheal LPS ARDS models [197,198]. These protective effects may be mediated by a reduction in pro-inflammatory proteins, tumor necrosis factor receptor-associated factor-6 (TRAF6) and IL-1 receptor-associated kinase 1 (IRAK1), which are possible targets of miR-146 [198]. The therapeutic-induced overexpression of miR-146 in ARDS patients may, therefore, inhibit the excessive inflammatory response characteristic of ARDS.

The study conducted by Xu demonstrated that the expression of miR-144-3p is elevated in sepsis-induced ALI, both in patients and in mouse models. A further evaluation of miR-144-3p in mice led to the discovery that it acts through the inhibition of Caveolin2, thus activating the JAK/STAT pathway and increasing the production of pro-inflammatory factors and cell apoptosis [199]. The downregulation of mir-19a-3p was found to improve inflammation and ALI induced by sepsis in both in vivo and in vitro experiments, as the reduction in pro-inflammatory cytokines demonstrated. It seems that the target gene of miR-19a-3p is represented by USP13, whose over-expression is related to amelioration and better outcomes of lung injury [200].

Another study focused on the role of miR-199a, demonstrating its involvement in increasing the production of pro-inflammatory factors, thus playing a role in the pathogenesis of ARDS. In the same study, a miR-199a antagomiR markedly reduced the pro-inflammatory cytokines in lung macrophages and septic lung tissues. Moreover, the activity of MPO, a biomarker for the function and activation of neutrophils, was also significantly reduced by miR-199a antagomiR in lung. The study also demonstrated that miR-199 operates via targeting and degrading SIRT1 [201]. An analogue mechanism was demonstrated for miR-34a [202]. Even miR-132 was found to provoke and worsen ALI through the inhibition of SIRT1, facilitating the expression of Bax [203]. MiR-30d-5p contained in exosomes of neutrophils showed the capacity to induce macrophage M1 polarization and trigger macrophage pyroptosis via activating the NF-κB signaling pathway, thus deteriorating lung injury [204]. Another exosomal miRNA is miR-1298-5p. It was found to be markedly increased in blood collected from septic patients with lung injury. Ma et al. evaluated its role in rat models, demonstrating that miR-1298-5p is responsible for augmented cell permeability and enhanced inflammatory reaction, thus triggering bronchial epithelial cell injury. It exerts those effects through the downregulation of SOCS6 and promoting the STAT3 pathway [163].

Recently, miR-92A-3p resulted in increased levels of intrapulmonary inflammation and oxidative stress by silencing AKAP1 in mice [205].

Other miRNAs have also been found to play a protective role, thus improving or even preventing lung injury in sepsis. One of them is miRNA-23 a, whose role was studied by Yang et al. They conducted both in vitro and in vivo experiments, finding out that miRNA-23 overexpression improves sepsis-induced lung injury via inhibiting PTEN and suppressing P53, and also by stimulating the PI3K/AKT pathway [206].

Even miR-218 seemed to determine a reduction in inflammation in both in vitro and in vivo models by inhibiting the activity of the NF-κB pathway, thus lowering levels of pro-inflammatory cytokines such as TNF-α, IL-1β, and IL-6 [115].

MiR-539-5p, miR-145, and miR-124 were also related to the improvement of ALI induced by sepsis, despite acting through different molecular pathways. In particular, miR-539-5p seems to decrease the expression of pro-inflammatory cytokines, IL-1β and IL-6, and suppress the transcriptional activity of apoptosis marker, caspase-3 via targeting and suppressing ROCK1. Such results were obtained both in vivo (in CLP-operated mice) and in vitro (in LPS-treated MPVECs) models [207].

The work conducted by Cao showed that miR-145 inhibited LPS-induced inflammation and sepsis-induced lung injury via directly targeting TGF-β2 and inactivating TGFBR2/ Smad3 signaling both in septic patients and mice [139]. They also found that miR-21 levels appeared to be downregulated in septic patients, which countered previous works showing that miR-21 levels are upregulated in septic patients. They explain such a result by the fact that a different blood source, from late-stage sepsis patients, was chosen in previous work, indicating that miR-21 levels might be different in different stages of sepsis. Moreover, in their study, a protective role was demonstrated for miR-155, in contrast to the aforementioned studies.

In Pan’s work, it was demonstrated that miR-124 inhibited the activation of the MAPK pathway by inhibiting the expression of MAPK14, so as to reduce the severity of septic shock in ALI mice [208]. Indeed, the overexpression of miR-124 or the silencing of MAPK14 could downregulate the expression of inflammatory cytokines, both those that induce (TNF-α, IL-6, IL-1β) and those that inhibit inflammation (IL-10) in ALI mice, thus inhibiting or limiting the degree of tissue damage and pulmonary edema due to an inflammatory response.

On the contrary, miR-483-5p and miR-497-5p resulted in aggravating pulmonary disease induced by sepsis since their overexpression determined an increased production of TNFα, IL1β and IL6, thus promoting inflammation. They also increased the activation of Caspase-3, favoring cell apoptosis. Mir-483-5p exerts its role by targeting PIAS1. Moreover, the authors of the study suggested that mir-483-5p could be used as a potential prognostic marker of sepsis [209]. miR-497-5p, though, acts through the downregulation of IL2RB [210].

The role of miR-802 was evaluated by You [211], who showed how miR-802 alleviates inflammation in sepsis-induced ALI; its upregulation can reduce the production of pro-inflammatory cytokines, but, on the other hand, it is unable to inhibit cell death induced by LPS. The study also found that miR-802 acts via inhibiting Peli2, a positive regulator in the LPS/TLR4 pathway, and also NFκB. MiRNA-326 seemed to prevent inflammation through the inhibition of TLR4, as well [212].

MiR-129-5p and miR-490 were found to reduce inflammation in lungs of CLP mice. Indeed, they have been shown to reduce levels of pro-inflammatory factors and even reduce apoptosis. However, the molecular pathways behind such effects remain unclear [213,214]. More recently, miR-129 was discovered to exert its protective role by blocking the TAK1 and NF-κB pathways [215].

Xie et al. showed a significantly reduced expression of miR-127 during lung injury in vivo, while the administration of miR-127 probed attenuated pulmonary inflammation through the regulation of CD46 in macrophages [216].

An important contribution was provided by Jiang, who discovered that exosomal miR-125-5p alleviates sepsis-induced lung injury via suppressing topoisomerase II alpha. Specifically, in this study, the overexpression of miR-125-5p allowed reduced levels of pulmonary oedema, inflammation, and apoptosis in rats with induced ALI [217]. Even miR-499-5p showed similar functions in the work of Zhang et al., but via targeting and degrading Sox6 [218].

The same results were obtained by Lu, who evaluated the role of miR-942-5p, but through in vitro experiments, thus demonstrating the effects of reduction in inflammation and apoptosis only. Thanks to their experiments, they demonstrated that miR-942-5p exerts its effect by targeting and inhibiting TRIM37 [219].

Finally, miR-16-5p was demonstrated to reduce sepsis-induced inflammation, apoptosis, and lung oedema in in vivo models. MiR-16-5p acts by inhibiting BRD4, but it is, in turn, inhibited by lncRNA NEAT1 [220].

Other studies have demonstrated that the contribution of miRNAs in sepsis is regulated by lncRNA. For example, the action of miR-34b-5p was found to be blocked by lncRNA TUG1, where the overexpression of TUG1 led to the attenuation of inflammation and a reduction in apoptosis [221]. Such effect implicates that miR-34b-5p is usually overexpressed in sepsis and in sepsis-induced ALI. It seems that miR-34b-5p performs its role via inhibiting Gab1, a protein implicated in the production of surfactant protein in alveolar type II cells. Moreover, miR-195-5p, which is generally involved in inducing and worsening ALI triggered by sepsis through inhibition of PDK4, could be stopped and downregulated by lncRNA CASC9 [222]. This leads to improvements in sepsis-induced lung injury. Another miRNA involved in the PDK4 pathway is miR-152-3p, which was found to be blocked by lncRNA CASC2 in vitro. Even in this case, such blockade resulted in the resolution of inflammation and lung injury induced by exposure to LPS [223]. Another lncRNA with a protective role is OIP5-AS1, which inhibits miR-128-3p, thus enabling the activation of the SIRT1 signaling pathway [224].

Another miRNA controlled by lncRNA is miR-424, downregulated by THRIL. MiR-424 is negatively associated with inflammation; thus, its inhibition results in the overexpression of ROCK1 and, consequently, increased apoptosis and worsened lung injury [225]. miRNA expression in sepsis-related lung dysfunction are summarized in Table 4.

#### 3.3.5. miRNA Expression in Sepsis-Related Liver Dysfunction

The liver is a critical point for numerous physiological processes, for example, macronutrient metabolism, blood volume control, immune system support, and lipid and cholesterol homeostasis.

Liver is also an important organ for host defense homeostasis and represents a target tissue that is frequently damaged by sepsis, making liver injury a common complication of sepsis [228]. However, the full mechanism of liver injury in sepsis is still unclear.

The following papers represent the most recent studies investigating changes in miRNA expression in liver tissue during sepsis.

Yang et al. found that miR-155 expression was upregulated in the liver tissue of septic mice. The administration of miR-155 antagomiR reduced septic liver injury with the inhibition of oxidative stress, cell apoptosis and mitochondrial dysfunction via targeting Nrf-2 [229].

Ling and colleagues studied liver tissue from septic rats and found low miR-30a levels, the increased expression of FOSL2 (fos-related antigen-2) and the activation of the JAK/STAT pathway, suggesting possible involvement in apoptosis and the proliferation of septic rat hepatocytes [230].

Yuan et al. instead described miR-30a upregulation and the downregulation of SOCS1 (suppressor of cytokine Signaling protein 1) via the JAK/STAT pathway in rat sepsis liver tissue, with decreased liver cell proliferation and increased hepatocyte apoptosis [231].

Zhu et al. reported low miR-98 levels in liver, heart, and lung of septic mice compared to controls. Further investigation revealed that miR-98 upregulation in turn downregulated pro-inflammatory mediators (IL-6 and TNAF-α) and upregulated anti-inflammatory mediators (IL-10) in all three tissues of septic mice. This study showed that miR-98 could protect the liver (but also heart and lung) from septic injury by negatively mediating HMGA2, which may be related to the inhibition of the NF-κB pathway [86].

Zhou and Xia reported high levels of miR-103a-3p in the liver and serum of LPS septic mice and also in human patients. In vitro miR-103a-3p inhibition suppressed LPS-induced inflammation by downregulating the expression of TNF, IL-1β and IL-6 in vitro and in vivo [232].

Other studies came to different conclusions. Chen et al. described low miR-103 levels in the liver tissue of septic mice compared to controls. Researchers also found that miR-103a-3p agomiR was related to decreased septic liver injury with the suppression of inflammatory response and cell apoptosis targeting HMGB1 (high-mobility group B1), which is involved in the induction of inflammation and multiple organ failure in acute liver failure [233].

Similar results were found by Li et al. who reported decreased miR-103a levels in liver (and also lung) of LPS septic mice compared to controls. Li also analyzed blood samples from septic human patients, characterized by low miR-103a-3p expression compared to healthy people. In addition, Li found that miR-103a-3p binds specifically to HMGB1, contributing to its downregulation [234]. Both studies suggest that miR-103a could have a protective role in liver tissue during sepsis, leading to an attenuation of the inflammatory response.

Gu and colleagues described low miR-425-5p levels in the liver tissue of LPS septic mice, along with high liver injury. Moreover, researchers found that miR-425-5p overexpression protects against LPS-induced necrosis and inflammatory responses by inhibiting RIP1 (receptor interacting serine/threonine kinase 1) [235].

Zhou et al. analyzed the effect of miR-10a using liposome transfection in septic rats and found significantly increased miR-10a levels in the liver tissue of the mimic group and significantly decreased levels in the inhibitor group. The silencing of miR-10a can inhibit sepsis-induced liver injury in rats, suggesting a possible protective role, with the downregulation of the TGF-β1/Smad pathway and the inhibition of the expression of IL-6, ROS and TNF controlling inflammatory development in septic rats [236].

Xu et al. showed that miR-142-5p expression was significantly upregulated in the liver tissue of septic mice (and also in LPS-induced hepatocytes) compared to controls. miR-142-5p upregulation could suppress hepatocyte survival by enhancing hepatocyte inflammation and apoptosis. Experimental data also showed that the inhibition of miR-142-5p upregulated SOCS1 in septic mice [237].

Kim et al. described high miR-147 levels in the liver tissue of a septic mouse model compared to controls. miR-147 was also induced in the stomach, lung, and kidney, but decreased in the brain [238]. miR-147 seems to be involved in cell cycle regulation, but also in the inflammatory response.

Li et al. showed reduced miR-373-3p levels in sepsis-induced liver injury models, while the administration of miR-373-3p mimics promoted the viability and reduced apoptosis of THLE-3 cells treated with LPS, implying that miR-373-3p plays a protective role in sepsis. miR-373-3p is in turn regulated by lncRNA LINC00472, with levels found to be upregulated in LPS-induced acute liver injury models [239].

Another miRNA that is regulated by lncRNA is miR-126-5p, the role of which has been studied in rat models of sepsis and also in L02 cells transfected with LPS. miR-126-5p was upregulated in sepsis and its increase was associated with augmented apoptosis and decreased cell viability. miR-126-5p is targeted by the lncRNA CRNDE, which in turn is downregulated in liver tissue and cells during sepsis. Such results have been shown both in vivo and in vitro [240]. Another lncRNA involved in LPS-induced liver injury is HULC, whose levels were elevated in the serum of septic patients. As with other lncRNAs, HULC can also regulate the expression of miRNAs, particularly miR-204-5p, which was downregulated in septic patients. A decrease in miR-204-5p has been associated with a worsening of LPS-induced liver injury [241].

Han et al. showed low miR-9 levels in sepsis as a result of MCPIP1 overexpression. MCPIP1 is a deubiquitinase and RNase, raised in septic mouse models, with a protective role in sepsis-induced liver injury. Interestingly, this protective function was only observed in the liver and not in other organs, suggesting that MCPIP1’s protective role is organ-dependent [242].

Another interesting study is that of Yang et al., which demonstrated that paclitaxel reduces LPS-induced liver injury, as its administration reduced the effects of necrosis, edema and neutrophil infiltration in LPS septic mice. Such effect was exerted through miRNA modulation, particularly by miR-27a upregulation. The overexpression of miR-27a ameliorates liver injury by inhibiting the NF-κB pathway [243].

In contrast, miR-640 exerts a damaging role in the liver of septic mice. Wang et al. demonstrated that miR-640 inhibition leads to a reduction in hepatocyte apoptosis and prevents excessive damage to liver structures, resulting in improved liver function [244].

miRNA expression in sepsis-related liver dysfunction are summarized in Table 5.

#### 3.3.6. miRNA Expression in Sepsis-Related Kidney Dysfunction

Critically ill patients with sepsis are prone to multi-organ failure (MOF), which is often associated with acute kidney injury (AKI) [245]. According to the main criteria of RIFLE and AKIN, the diagnosis of AKI is essentially based on the measurement of serum and urine creatinine levels and their persistence over time. If the persistence of increased serum creatinine levels and/or decreased urinary creatinine levels lasts longer than 7 days, we call it acute injury (AKI), and if this impairment lasts longer than 90 days, we speak of acute disease (AKD) [246]. Complications of AKI include hyperkalemia, uremia, fluctuations in fluid/electrolyte balance, and metabolic acidosis [247]. Epidemiologically, it has been calculated that the incidence of sepsis and severe sepsis is 31.5 million and 19.4 million, respectively, and the incidence of AKI in these patients can be as high as 47.5% [248], with a mortality rate of nearly 60%. Although the pathophysiology of AKI in sepsis is not fully understood, it is multifactorial and involves several immunologic aspects. Overall, septic AKI is associated with the onset of circulatory changes resulting in endothelial dysfunction and damage due to ischemia and reperfusion, the activation of an abnormal inflammatory cascade, oxidative radical production, mitochondrial dysfunction, immune suppression, and cellular changes with the activation of apoptosis and autophagy [249]. Several studies have shown that this disproportionate inflammatory response produces exaggerated levels of cytokines, leading to the formation of intravascular microthrombi with consequent oxygen deprivation. It appears that the Toll-like receptor family (TLR) in the membrane of tubule cells plays an important role in the uncontrolled activation of adaptive and innate immunity. Indeed, TLRs (TLRs -1, -2, -3, -4, and -6) present on the tubule cell membrane activated by endotoxins recognize the molecular patterns associated with pathogens (PAMPs) and damage (DAMPs) and stimulate leukocyte activity, triggering the inflammatory cascade [250,251]. In septic AKI, the most important receptor appears to be TLR -4 on the tubule cell membrane because it can bind to the lipopolysaccharide endotoxin LPS. The activation of TLR-4 leads to the dysregulation of tubular integrity, resulting in the disruption of the tight junction. The disruption of these multiprotein complexes could, therefore, contribute to renal dysfunction with the clinical occurrence of oliguria.

In recent years, biomedical research has focused on identifying innovative biomarkers that can be used alongside traditional markers (serum and urine creatinine) in the diagnosis of AKI, such as neutrophil gelatinase-associated lipocalin (NGAL), cystatin C, kidney injury molecule-1 (KIM -1), interleukin 18 (IL-18), urinary insulin-like growth factor-binding protein-7 (IGFBP-7), TIMP-2 (tissue inhibitor of metalloproteinase 2), calprotectin, urinary angiotensinogen, and liver fatty acid-binding protein. One of the most promising research directions in this area is the use of non-coding RNAs (ncRNAs) as markers. NcRNAs are small nucleotide RNA molecules that are not translated into proteins but are involved in numerous biochemical and pathophysiological processes such as epigenetic regulation, cell cycle, development, and differentiation. Among ncRNAs, the most studied biomarkers in various physiological and pathological processes are microRNAs (miRNAs) and endogenous single-stranded non-coding RNA molecules that consist of few nucleotides (18-31), as these are abundant and expressed in a tissue-specific manner [252].

Wei et al. were the first to observe and demonstrate the active role of miRNAs in the pathogenesis of AKI in a Dicer knock-out model, i.e., in a mouse in which the gene DICER, which produces the enzyme ribonuclease III, was selectively knocked out. These mice showed normal renal function and at the same time increased resistance to externally induced AKI, with less tissue damage and less tubular apoptosis than control mice. The analysis of miRNAs in the renal cortex showed the global downregulation of miRNAs in these rats [253]. During sepsis, miRNAs were observed to be mainly involved in the activation of TLR-4, leading to the production of pro-inflammatory cytokines (IL-6, IL-1, TNF) that cause endothelial dysfunction and organ damage.

The pathogenesis of AKI in sepsis is challenging, and many miRNAs are likely involved in the pathophysiology and biochemical pathways.

Xu et al. identified 31 frequently differentially expressed miRNAs that play a role in the mechanism of sepsis-induced AKI. They preliminarily found 2 upregulated and 29 downregulated miRNAs in human blood patients with septic AKI compared with healthy controls. They investigated the effects of LPS on renal tissues and showed that it induced the downregulation of miR-15a-5p and the upregulation of XIST (X-inactive-specific transcript) and CUL3 (cullin 3 gene) with the inhibition of animal podocyte growth [254].

One of the most studied miRNAs in acute kidney injury during sepsis is miR-21 because of its role in regulating inflammation and energy metabolism. Fu et al. described the anti-apoptosis effects of miR-21 overexpression in renal tissues damaged by sepsis compared with the control group in vitro and vivo. Moreover, they observed that the upregulation of miRNA-21 during renal cell injury could regulate the PTEN/PI3K/AKT signaling pathway, which would have a protective effect and inhibit cellular apoptosis [255]. However, Wei et al. demonstrated the opposite effect: miR-21 silencing promoted renal function and inhibited cell apoptosis in septic mice, decreasing LPS-stimulated human renal cells [256]. Lin et al. observed how the upregulation of miR-21-3p affects the AKT/CDK2-FOXO1 pathway and causes cell cycle arrest and apoptosis, resulting in severe damage to the renal tubule epithelium [257]. Wei et al. also showed that the expression of miR-21e5p is upregulated in LPS-stimulated HK-2 cells and septic patients with AKI [258].

A previous study suggests that miR-21a-3p may play a crucial role in the kidney during sepsis by acting as an inflammatory mediator and manipulating tubular epithelial cell (TEC) metabolism. Zhiqiang Zou et al. attempted to elucidate this mechanism by analyzing septic rats and the TEC line. They showed that miR-21a-3p levels in plasma and TECs increased during sepsis by the internalization of plasmatic Ago2, which binds miR-21a-3p mediated by membrane Nrp-1 [259].

While the anti-apoptosis role of miR-22-3p is clear in various tumor cell lines [260], its role in septic AKI remains unclear. Pan Zhang et al. showed that the overexpression of miR-22-3p acts as a protective factor in human renal tubule epithelial cells (HK-2) treated with LPS to stimulate septic AKI. miR-22-3p, targeting the apoptosis-inducing factor mitochondrion-associated 1 (AIFM1) gene, inhibits renal cell apoptosis and reduces the accumulation of cleaved caspase-3 [261].

Xudong Wang and colleagues developed a sepsis–AKI model in rats in vivo and in HK-2 cells in vitro and analyzed the expression of miR-22-3p and PTEN. They demonstrated that miR-22-3p was significantly downregulated during septic AKI both in vivo and in vitro. PTEN is the target of this miRNA, and the overexpression of miR-22-3p inhibits the inflammatory response and cell apoptosis, exerting a protective role in sepsis-induced acute kidney injury [262].

Junwei Ye et al. observed the downregulation of miR-23a-3p expression both in vivo in septic AKI patients compared with healthy controls and in vitro in LPS-treated HK-2 cells. They showed that the overexpression of this miRNA inhibited Wnt/β-catenin signaling in LPS-treated HK-2 cells and increased renal cell function [263].

Yanhong Chen and colleagues identified the pathway responsible for the upregulation of miR-26a-5p during septic AKI. They demonstrated that sepsis induced by LPS treatment led to the activation of NF-κB in renal cells, resulting in the overexpression of miR-26a-5p. The effect of this miRNA appeared to be the silence of the pro-inflammatory IL-6 gene. Indeed, blocking miR-26a-5p promoted renal inflammation and exacerbated renal injury [264].

Zong-Lan Ha et al. determined the role of miR-29b-3p in sepsis-induced AKI and investigated the underlying mechanism. They demonstrated that the downregulation of miR-29b-3p in septic mice with AKI exacerbated podocyte injury by targeting the HDAC4 gene [265].

Gaihong Ding and colleagues conducted a study to investigate the inhibitory effect of miR-103a-3p on inflammatory and apoptotic signaling pathways in septic mice. They also investigated the molecular mechanisms of this miRNA in LPS-stimulated HK-2 cells and showed that miR-103a-3p inhibited inflammation and apoptosis via the pro-inflammatory gene CXCL12 pathway [266].

In an experimental hybrid study of 50 septic patients, miR-106a was overexpressed, resulting in THBS2 gene overexpression with the exacerbation of LPS-induced inflammation and the apoptosis of TCMK-1 cells [267].

Wang et al. recruited 15 septic AKI patients, 15 non-septic AKI patients, 15 septic non-AKI patients, and 15 healthy volunteers to investigate whether endothelial cells can directly cause tubular cell injury in septic AKI and the role of miRNA in this mechanism. They discovered that the overexpression of miR-107 stimulates TNF-α secretion by targeting DUSP7 in endothelial cells. This signaling pathway may explain renal cell injury in septic AKI because the inhibition of miR-107 attenuates TNF-α secretion and avoids the resulting tubule cell injury [268].

Huazhong Zhang observed the association between miR-124-3p.1 and the activity of lysophosphatidylcholine acyltransferase3 (LPCAT3), a key enzyme of phospholipid metabolism related to ferroptosis, in septic AKI. They demonstrated that the activity of LPCAT3 was significantly increased in the serum of patients with early SA-AKI, whereas miR-124-3p.1 was downregulated [269].

Lang Shi and colleagues identified the MEKK3/JNK pathway as a target of miR-150-5p, eliciting a protective effect against LPS-mediated HK-2 cell apoptosis. During AKI with sepsis, Stat-3 activation mediated the decrease in miR-150-5p, which enhanced renal injury [270].

Recent studies have shown that miR-155, among other miRNAs, is involved in the regulation of inflammatory processes and contributes to the pathological process of kidney injury via the regulation of innate and adaptive immune responses [271]. Yuqian Ren et al. showed that miR-155 is associated with the inflammatory pathway JAK/STAT and its inhibition can protect septic mice from kidney damage [272].

Yongjun Lin, et al. analyzed the expression patterns of miR-210, miR-494, and miR-205 in the blood of 110 patients with sepsis-related AKI and examined the association of these miRNAs with biochemical indices of sepsis and patient prognosis. The authors’ study suggests that upregulated miR-210 and miR-494 and downregulated miR-205 have predictive value for the prognosis and survival of patients with sepsis-induced AKI [273].

Zhenzhen Sang et al. showed that the overexpression of miR-214 during sepsis-induced AKI can ameliorate renal injury by reducing oxidative stress and inhibiting autophagy through the regulation of the PTEN /AKT/mTOR pathway [274].

Chen Guo and colleagues investigated the role of miR-214-5p in vivo and in vitro. They found that the inhibition of miR-214-5p reduced LPS-induced renal inflammation and oxidative stress, thereby preventing renal damage and dysfunction. In contrast, the stimulation of miR-214-5p aggravated LPS-induced inflammation, oxidative stress, and AKI in vivo and in vitro. The researchers concluded that the inhibition of miR-214-5p prevented septic AKI via the activation of AMPK and that compound C treatment completely abrogated the renoprotective effect in mice [275].

In another recent study, Liu et al. investigated the role of miR-452 as an effective biomarker for acute kidney injury in sepsis induced by LPS in mice. They showed that the overexpression of miR-452 stimulated the pro-inflammatory NF-κB pathway both in vitro and in vivo. Interestingly, they found the overexpression of miR-452 in the serum and urine of mice in the early phase of septic AKI, even before detectable renal injury [276].

Qi et al. instead analyzed miR-665 expression in rats with septic acute kidney injury. They detected the overexpression of miR-665 in the renal tissues of septic rats with AKI and found that the downregulation of miR-665 could inhibit LPS-induced renal inflammation and apoptosis and improve renal function [277].

Qin-Min Ge et al. identified specific miRNAs involved in sepsis-induced AKI in the blood serum of human patients and investigated their target pathways. They found that miR-4321 and miR-4270 were statistically significantly upregulated in sepsis-induced AKI compared with non-sepsis AKI. Pathway analysis revealed that several significant signaling pathways of the predicted target genes were associated with oxidative stress. miR-4321 was involved in regulating the expression of AKT1, mTOR, and NOX5, whereas miR-4270 was involved in regulating the expression of PPARGC1A, AKT3, NOX5, PIK3C3, and WNT1 [278].

In a 2016 study, circulating miR-192-5p was observed to discriminate between patients with sepsis and those with an active, non-infectious inflammatory response in critically ill patients [279]. A subsequent study by Zou in 2017 showed that miR-192-5p expression increases in the urine of mice in which AKI is induced after ischemia reperfusion [280]. Further studies on this molecule may, therefore, reveal its correlation with septic AKI.

The following table summarizes recent results about miRNA expression during sepsis-induced AKI.

miRNA expression in sepsis-related kidney dysfunction are summarized in Table 6.

## 4. Discussion

In recent years, miRNAs have taken a prominent role in the study of non-coding RNAs [281]. Many studies have focused on the role of miRNAs in the pathophysiology of various diseases such as cancer, and neurodegenerative and immune-mediated diseases [282,283,284,285,286]. As knowledge has advanced, it has become apparent that the most important targets of miRNAs are the genes that regulate the complicated inflammatory process [287,288,289]. Given that one of the major critical problems in sepsis is the dysregulated host inflammatory response to infection, the existence of numerous miRNAs involved in the multi-organ dysfunction caused by sepsis should not be a surprise.

A literature review of miRNAs involved in individual organs (brain, heart, lung, liver, kidney, and blood) during sepsis revealed that the most frequently mentioned target genes and pro-target AKTs (serine/threonine kinases), FOXs (forkhead box), and HMGs (high-mobility groups) are NF-κB (nuclear factor kappa B), SIRT (sirtuin), SOCS (suppressor of cytokine signaling), STAT (signal transducer and activator of transcription), TRAF (TNF receptor-associated factor), and most importantly, PTEN (phosphatase and tensin homolog).

Some of these genes or proteins have long been known to be biological mediators of inflammatory processes, such as HMGB1 [290], NF-κB [291], SOCS [292], TRAF [293], and PTEN [294]. Interestingly, however, genes or proteins involved in cell cycle regulation, metabolism, and stress response are also involved in this complicated disease mechanism.

For example, AKT protein is a cytosolic protein that plays a key role in the PI3K/AKT/mTOR pathway by determining cell growth and resistance to apoptosis [295]. On the other hand, FOX proteins regulate the expression of genes involved in cell growth, proliferation, differentiation, and longevity, and it is not surprising that they have been implicated in tumor growth [296,297,298]. SIRT is a protein encoded by the gene of the same name that has the ability to regulate cell metabolism in response to stressors through epigenetic silencing [299]. Finally, STAT mediates the expression of a variety of genes involved in cellular responses to different stimuli and plays a key role in cell growth and apoptosis [300].

Therefore, these findings may further deepen and complicate the biomolecular study of sepsis, as not only the classical violent inflammatory response triggered by infection may be involved in organ dysfunction, but also intrinsic genetic traits of each individual involved in cellular regulation. Given the properties of miRNAs, as small molecules that are very stable and conserved in the human species, we should direct current and future research towards this class of ncRNAs to better understand sepsis.

However, the use of miRNAs as diagnostic and prognostic biomarkers in clinical practice is currently limited due to several issues. MiRNAs can be detected by various approaches, such as Northern blotting, microarray, next-generation sequencing (NGS), and real-time PCR; however, each of these approaches has some limitations. Microarray and Northern blotting indeed have low sensitivity or specificity and require relatively large amounts of RNA. Real-time quantitative PCR (RTq-PCR) is currently the most used method because it has high sensitivity and specificity. However, it is time consuming and requires the use of primer sequences. In addition, its cost is relatively high. Finally, NGS is also a technique with high sensitivity and sensitivity. In addition, it does not require primers for its application and allows the detection of all miRNAs present in a sample [301,302,303]. Such limitations arise from the fact that the field of miRNAs is still largely unexplored. Consequently, a standardized method for their identification and measurement has not been fully developed. However, it is possible that these obstacles can be overcome in the foreseeable future so that miRNAs become efficient diagnostic, prognostic, and therapeutic tools that can be readily applied in daily clinical practice.

Below are overview tables [Table 7, Table 8, Table 9, Table 10, Table 11 and Table 12] showing the miRNAs studied, their signaling pathways, and their effects on each organ. 

## 5. Conclusions

With this work, we aimed to collect and unify the current knowledge on the use of miRNAs as markers of sepsis to elucidate the biological processes that regulate this pathological process. miRNAs are molecules that have increasingly attracted the attention of scientists for decades due to their fundamental role in regulating biological processes, including inflammation during sepsis. Our study has shown that the major targets of miRNAs during sepsis appear to be not only inflammation-related genes or proteins, but also those related to cell cycle regulation and metabolism. In many cases, it was observed that certain miRNAs can be used as diagnostic or predictive markers for subsequent clinical outcome. In some cases, it was also observed how their pharmacological regulation could lead to tangible therapeutic benefits. Our results indicate that our biological and molecular understanding of sepsis is still at an early stage. Therefore, further thorough studies on this topic may allow the development of new early diagnostic and selective therapeutic methods.

## Figures and Tables

**Table 1:** miRNA expression in sepsis-related brain dysfunction.

Author and Reference	Year of Publication	Sepsis Model	Brief Description of miRNAs in Sepsis-Induced Brain Injury
Dong et al. [19]	2019	In vivo	miR-181b is downregulated in hippocampus
Yu et al. [21]	2019	In vitro	miR-200a-3p upregulation induced inflammatory response in sepsis-induced brain injury
Chen et al. [20]	2020	In vivo	miR-181b is overexpressed in septic cerebral cortex
Visitchanakun et al. [22]	2020	In vivo	miR-370-3p overexpression in SAE (in brain tissue and blood) miR-370-3p associated with TNF-α and increased brain apoptosis in SAE mice
Kim et al. [23]	2021	In vivo	miR-147 is downregulated in brain compared to other organ and also compared to controls
Nong et al. [24]	2021	In vivo	miR-126 downregulation is related to a protective role against sepsis-related blood–brain barrier damage
Rani et al. [25]	2022	In vivo	miR-190a-3p, let-7a-1-3p, miR-3085-3p were overexpressed in young and older females on day 1; miR-383-5p was instead downregulated in young males and females on day 4
Zou et al. [26]	2022	In vivo In vitro	miR-146a induces CXCL2 in microglia and reduces CXCL” in astrocytes miR-146a knock-out is related to low levels of neutrophils and monocytes in brain tissue

**Table 2:** miRNA expression in sepsis-related heart dysfunction.

Author and Reference	Year of Publication	Sepsis Model	Brief Description of miRNAs in Cardiac Tissue during Sepsis
Wang et al. [54]	2014	In vivo	mir-223 was downregulated; downregulation increased inflammation and myocardial injury
Liu et al. [56]	2020	In vivo	miR-223 was upregulated; upregulation reduced inflammation and apoptosis
M’baya-Moutoula et al. [57]	2018	In vitro	miR-223 expression regulates NF-κB pathway
Xue et al. [58]	2015	In vivo	miR-27a was upregulated; upregulation increased inflammation
Gao et al. [60]	2015	In vivo	miR-146a was upregulated; upregulation reduced inflammation and myocardial injury
An et al. [61]	2018	In vitro	miR-146a was upregulated; upregulation reduced inflammation and myocardial injury
Xie et al. [62]	2019	In vivo	miR-146a upregulation reduced inflammation and myocardial injury
Wang et al. [63]	2018	In vivo	miR-146b was upregulated; upregulation reduced inflammation and myocardial injury
Ma et al. [64]	2016	In vivo In vitro	miR-125b was downregulated and its upregulation reduced inflammation and myocardial injury
Wei et al. [65]	2019	In vitro	miR-150-5p was downregulated and its upregulation reduced inflammation and myocardial injury
Zhu et al. [66]	2020	In vivo In vitro	miR-150-5p was downregulated and its upregulation reduced inflammation and myocardial injury
Wang et al. [67]	2016	In vivo	miR-21-3p was upregulated and its downregulation reduced inflammation and cardiac disfunction
Wang et al. [68]	2016	In vivo	miR-155 was upregulated and its downregulation reduced inflammation and myocardial injury
Zhou et al. [71]	2017	In vivo	miR-155 was upregulated and overexpression reduced inflammation and myocardial injury
Diao et al.. [69]	2017	In vivo	miR-124a was downregulated and its upregulation reduced inflammation and myocardial injury
Zheng et al. [70]	2017	In vivo	miR-135a was upregulated; upregulation increased myocardial inflammation
Ge et al. [72]	2018	In vivo	miR-214 was upregulated; upregulation reduced inflammation and myocardial injury
Sang et al. [73]	2020	In vivo	miR-214-3p was upregulated; upregulation reduced inflammation and myocardial injury
Fang et al. [74]	2018	In vivo In vitro	miR-874 was upregulated; upregulation increased inflammation
Tang et al. [78]	2018	In vitro	miR-93-3p was downregulated and its upregulation reduced inflammation and myocardial injury
Yao et al. [79]	2018	In vivo In vitro	miR-25 was downregulated and its upregulation reduced inflammation and myocardial injury
Wu et al. [80]	2018	In vivo In vitro	miR-494-3p was downregulated and its upregulation reduced inflammation and myocardial injury
Zhang et al. [81]	2018	In vivo In vitro	miR-23b was upregulated; upregulation reduced inflammation and myocardial injury
Cao et al. [84]	2019	In vivo In vitro	miR-23b was upregulated; upregulation reduced inflammation and myocardial injury
Guo et al. [85]	2019	In vivo	miR-495 was downregulated and its upregulation reduced inflammation and myocardial injury
Zhu et al. [86]	2019	In vivo	miR-98 was downregulated and its upregulation reduced inflammation and myocardial injury
Ouyang et al. [87]	2020	In vivo	miR-208-5p was upregulated; upregulation increased inflammation and determined myocardial injury
Sun et al. [88]	2020	In vivo	miR-328 was upregulated; upregulation increased inflammation
Zhu et al. [91]	2020	In vivo	miR-29a was upregulated; upregulation increased inflammation and determined myocardial injury
Song et al. [92]	2020	In vivo In vitro	miR-29a was upregulated; upregulation reduced inflammation and apoptosis
Chen et al. [93]	2020	In vitro	miR-24 was downregulated; upregulation increased inflammation and determined myocardial injury
Sun et al. [94]	2020	In vivo In vitro	miR-192-5p was downregulated; downregulation reduced inflammation and cardiac disfunction
Wei et al. [95]	2020	In vitro	miR-144-3p was downregulated; upregulation reduced inflammation and myocardial injury
Xing et al. [96]	2020	In vivo In vitro	miR-330-5p was downregulated; upregulation reduced inflammation and myocardial injury
Han et al. [97]	2020	In vivo In vitro	miR-1-5p was downregulated; downregulation reduced inflammation and cardiac disfunction
Li et al. [98]	2020	In vivo	miR-29b-3p was downregulated; upregulation reduced inflammation and myocardial injury
Xin et al. [99]	2021	In vivo In vitro	miR-101-3p was upregulated; downregulation reduced inflammation and cardiac disfunction
Liu et al. [100]	2021	In vivo In vitro	miR-106b-5p was upregulated; upregulation reduced inflammation
Zhang et al. [101]	2021	In vivo	miR-29c-3p was upregulated; upregulation increased inflammation and determined myocardial injury
Yang et al. [102]	2021	In vivo In vitro	miR-499a-5p was downregulated; upregulation reduced inflammation and myocardial injury

**Table 3:** miRNA expression in blood during sepsis.

Author and Reference	Year of Publication	Sepsis Model	Brief Description of miRNAs in Blood during Sepsis
Wang et al. [113]	2019	In vivo	miR-494-3p was downregulated; upregulation reduced inflammation through reduction in TNF-α levels and reduction in NF-κB p65 nuclear translocation
Li et al. [114]	2018	In vivo In vitro	miR-218 was downregulated; upregulation reduced inflammation
Zhou et al. [115]	2018	In vivo	miR-218 was downregulated; upregulation reduced inflammation
Abou El-Khier NT et al. [116]	2018	In vivo In vitro	miR-122 was upregulated, and can be used as a prognostic biomarker for sepsis
Sun et al. [117]	2020	In vivo	miR-328 was upregulated; downregulation reduced inflammation. Its expression was a good diagnostic marker for sepsis
Na et al. [118]	2020	In vivo In vitro	miR-21 was downregulated, representing a good predictor for sepsis risk
Lin et al. [119]	2020	In vivo	miR-126 was upregulated, representing a good marker for sepsis
Li et al. [120]	2020	In vivo	miR-125a was upregulated, representing a good marker for sepsis
Song et al. [121]	2017	In vivo In vitro	miR-146a was upregulated, upregulation improved survival in septic mice. It represents a good biomarker and predictor for high sepsis risk
Chen et al. [122]	2020	In vivo	miR-146a was upregulated; upregulation was associated with increased inflammation. It represents a good biomarker and predictor for high sepsis risk
Bai et al. [123]	2018	In vivo	miR-146a was upregulated; upregulation improved survival in septic mice. It represents a good biomarker and predictor for high sepsis risk
Brudecki et al. [124]	2013	In vivo In vitro	miR-146a was upregulated; upregulation improved survival in septic mice. It represents a good biomarker and predictor for high sepsis risk
Banarjee et al. [125]	2013	In vivo In vitro	miR-146a was upregulated; upregulation improved survival in septic mice. It represents a good biomarker and predictor for high sepsis risk
Mohnle et al. [126]	2015	In vivo In vitro	miR-146a was downregulated; downregulation induced inflammation
Paik et al. [127]	2019	In vivo	miR-146a was downregulated; downregulation induced inflammation
Chen et al. [122]	2020	In vivo	miR-146b was upregulated; upregulation was associated with increased inflammation. It represents a good biomarker and predictor for high sepsis risk
Zhang et al. [128]	2020	In vivo	miR-146b was upregulated; upregulation reduced inflammation
Gao et al. [129]	2017	In vivo	miR-146b was upregulated; upregulation reduced inflammation
Zou et al. [130]	2020	In vivo	miR-126 was upregulated, representing a good marker for sepsis
Dang et al. [131]	2020	In vivo In vitro	miR-223 was expressed in sepsis; upregulation reduced severity of sepsis
Yao et al. [132]	2015	In vivo	miR-25 upregulation reduced oxidative stress
Xue et al. [133]	2019	In vivo In vitro	miR-21 was upregulated; downregulation reduced inflammation
Liu et al. [56]	2020	In vivo	miR-223 was downregulated; represents a good biomarker for sepsis
Benz et al. [134]	2015	In vivo	miR-223 serum levels not correlated with sepsis, either in mouse models or in human patients.
Wu et al. [135]	2018	In vivo	miR-223 was upregulated; upregulation was associated with disease severity and inflammation
Zhou et al. [136]	2016	In vivo In vitro	miR-205-5b was downregulated with increased HMGB1 expression and inflammation
Wu et al. [137]	2020	In vivo In vitro	miR-145a was downregulated; upregulation reduced inflammation
Ma et al. [138]	2019	In vivo In vitro	miR-145a was downregulated; upregulation reduced inflammation
Cao et al. [139]	2019	In vivo In vitro	miR-145a was downregulated; upregulation reduced inflammation
Pan et al. [140]	2018	In vivo In vitro	miR-145a was downregulated; upregulation reduced inflammation
Zhao et al. [120]	2020	In vivo	miR-125b was upregulated; it is a marker of sepsis and risk of mortality
Sisti et al. [141]	2018	In vivo In vitro	miR-125b was upregulated; it is a marker of sepsis and risk of mortality
Zhu et al. [142]	2020	In vivo	miR-125b was upregulated; it is a marker of sepsis and risk of mortality
Sun et al. [143]	2021	In vivo In vitro	miR-27b was downregulated; upregulation reduced levels of inflammation
Sisti et al. [141]	2018	In vivo In vitro	miR-203b was downregulated; its expression reduced inflammation
Zheng et al. [144]	2020	In vivo In vitro	miR-10 a was downregulated; its expression was associated with disease’s severity scores
Liu et al. [145]	2015	In vivo	miR-155 was upregulated; upregulation was associated with severity of disease
Zhang et al. [146]	2019	In vivo	miR-7110-5p was upregulated, representing a good biomarker for sepsis
Jiang et al. [147]	2015	In vivo In vitro	miR-19a was upregulated, upregulation determined an increase in BCR signaling. Downregulation promoted the expression of CD22
Xu et al. [148]	2020	In vivo In vitro	miR-19b-3p was downregulated; upregulation reduced inflammation
Liu et al. [149]	2021	In vivo In vitro	miR-150 was downregulated; upregulation reduced inflammation
Sheng et al. [150]	2017	In vivo	miR-375 was downregulated; upregulation reduced inflammation
McClure et al. [151]	2014	In vivo	miR-21 and miR-181b were upregulated; downregulation reduced immunosuppression and improved bacterial clearance
McClure et al. [152]	2017	In vivo	miR-21 and miR-181b were upregulated; downregulation reduced immunosuppression and improved bacterial clearance
Han et al. [153]	2016	In vivo	miR-143 was upregulated, representing a good marker for sepsis
Liu et al. [154]	2020	In vivo In vitro	miR-20b-5p was upregulated; downregulation reduced inflammation
Ji et al. [155]	2019	In vivo In vitro	miR-17-5p was downregulated; upregulation reduced inflammation
Szilagyi et al. [156]	2020	In vivo	miR-26b was downregulated; downregulation increased inflammation and mortality
Chen et al. [157]	2020	In vivo In vitro	miR-96-5p was downregulated; upregulation reduced inflammation
Wang et al. [158]	2014	In vivo In vitro	miR-27a was upregulated; downregulation reduced inflammation and promoted survival
Yang et al. [159]	2018	In vivo	miR-27a was upregulated; downregulation reduced inflammation and promoted survival
Wang et al. [43]	2012	In vivo	miR-574-5p was upregulated; upregulation increased mortality rate
Sun et al. [45]	2012	In vivo	miR-181b was downregulated; upregulation reduced inflammation and mortality
Yang et al. [160]	2019	In vivo In vitro	miR-346 upregulation promoted proliferation
Bai et al. [161]	2020	In vivo In vitro	miR-148a-3p was upregulated; upregulation induced inflammation
Zhang et al. [162]	2019	In vivo In vitro	miR-218-5p was upregulated; downregulation improved survival
Ma et al. [163]	2021	In vivo	miR-1298-5p was upregulated; upregulation reduced inflammation
Gu et al. [164]	2020	In vitro	miR-608 was upregulated; upregulation reduced inflammation
Zhang et al. [165]	2018	In vivo	miR-124 was downregulated; upregulation reduced inflammation
Huo et al. [166]	2017	In vivo	miR-29a was upregulated, and is considered an independent risk factor for mortality in sepsis
Cao et al. [167]	2021	In vivo In vitro	miR-155 was upregulated; upregulation induced inflammation
Ma et al. [168]	2017	In vivo In vitro	miR-155 was upregulated; upregulation induced inflammation
Wang et al. [169]	2014	In vivo	miR-30a was downregulated; upregulation reduced inflammation
Mei et al. [170]	2019	In vivo In vitro	miR-339-5p was downregulated; upregulation reduced inflammation
Wang et al. [171]	2016	In vivo	miR-99b was upregulated
Yao et al. [172]	2020	In vitro	miR-215-5p was downregulated; upregulation reduced inflammatory response
Wang et al. [173]	2012	In vivo In vitro	miR-15a was upregulated, and is useful to distinguish sepsis from SIRS
Liang et al. [174]	2020	In vivo In vitro	miR-206 was upregulated, and is considered a good biomarker for sepsis
Wang et al. [175]	2020	In vivo	miR-92a-3p was downregulated; upregulation induced inflammation

**Table 4:** miRNA expression in sepsis-related lung dysfunction.

Author and Reference	Year of Publication	Sepsis Model	Brief Description of miRNAs in Lung Tissue during Sepsis
Liu et al. [191]	2017	In vivo In vitro	miR-155 was upregulated; upregulation attenuated inflammation in vivo and in vitro
Jiang et al. [192]	2019	In vivo	miR-155 was upregulated; upregulation induced inflammation and macrophage activation
Yuan et al. [193]	2016	In vivo	miR-155 was upregulated; upregulation induced inflammation. It was targeted by TREM-1
Li et al. [195]	2020	In vivo	miR-155 was upregulated; upregulation induced inflammation. Downregulation reduced inflammation
Han et al. [196]	2016	In vivo	miR-155 was downregulated
Vergadi et al. [197]	2014	In vivo	miR-146 was upregulated; upregulation protected against LPS-induced lung injury
Zeng et al. [198]	2013	In vivo	miR-146 was upregulated; upregulation protected against LPS-induced lung injury
Xu et al. [199]	2021	In vivo	miR-144-3p was upregulated; upregulation induced inflammation and apoptosis
Ren et al. [200]	2021	In vivo	miR-19a-3p was upregulated; upregulation induced inflammation
Liu et al. [201]	2018	In vivo	miR-199a was downregulated; downregulation reduced inflammation
Chen et al. [202]	2020	In vivo	miR-34a was downregulated; downregulation reduced inflammation
Qiu et al. [221]	2020	In vivo In vitro	miR-34b-5p was downregulated; downregulation reduced inflammation and lung injury
Zhu et al. [203]	2021	In vivo In vitro	miR-132 was upregulated; upregulation induced LPS-induced lung injury
Jiao et al. [204]	2021	In vivo	miR-30d-5p was upregulated; upregulation induced lung injury
Ma et al. [226]	2021	In vivo In vitro	miR-1298-5p was upregulated; upregulation induced inflammation. Downregulation reduced inflammatory response
Wang et al. [205]	2022	In vivo	miR-92a-3p was upregulated; upregulation induced LPS-induced lung injury
Yang et al. [206]	2018	In vivo In vitro	miR-23a was upregulated; upregulation reduced inflammation and lung injury
Zhou et al. [227]	2018	In vivo In vitro	miR-218 was upregulated; upregulation reduced inflammation and lung injury
Meng et al. [207]	2019	In vivo	miR-539-5p was upregulated; upregulation reduced inflammation and apoptosis
Cao et al. [139]	2019	In vivo In vitro	miR-145 was downregulated; downregulation induced LPS-induced inflammation
Pan et al. [208]	2019	In vivo	miR-124 was downregulated, downregulation induced LPS-induced inflammation
Leng et al. [209]	2020	In vivo In vitro	miR-483-5p was upregulated; upregulation induced inflammation. Downregulation reduced inflammation and apoptosis
Lou et al. [210]	2021	In vivo In vitro	miR-497-5p was upregulated; downregulation reduced inflammation and apoptosis
You et al. [211]	2020	In vivo In vitro	miR-802 was downregulated; upregulation reduced inflammation and apoptosis
Wang et al. [212]	2020	In vivo In vitro	miR-326 was downregulated, upregulation reduced inflammation and lung injury
Yang et al. [213]	2020	In vivo In vitro	miR-129-5p was upregulated; upregulation reduced inflammation and lung injury
Yao et al. [215]	2021	In vitro	miR-129 was downregulated; upregulation reduced inflammation and lung injury
Lin et al. [214]	2021	In vivo	miR-490 was downregulated; upregulation reduced inflammation and lung injury
Xie et al. [216]	2012	In vitro	miR-127 was upregulated; upregulation reduced inflammation and apoptosis
Jiang et al. [217]	2021	In vivo	miR-125-5p was downregulated; upregulation reduced inflammation and apoptosis
Zhang et al. [218]	2021	In vivo	miR-499-5p was downregulated; upregulation reduced inflammation and apoptosis
Lu et al. [219]	2021	In vitro	miR-942-5p was downregulated; upregulation reduced inflammation and apoptosis
Yin et al. [220]	2021	In vivo	miR-16-5p was downregulated; upregulation reduced inflammation and lung injury
Wang et al. [222]	2020	In vitro	miR-195-5p was upregulated; upregulation induced inflammation. Downregulation reduced inflammation and apoptosis
Zhu et al. [223]	2021	In vitro	miR-152-3p was upregulated; downregulation reduced inflammation
Xie et al. [224]	2021	In vivo In vitro	miR-128-3p was upregulated; upregulation induced inflammation and macrophage activation
Chen et al. [225]	2020	In vivo In vitro	miR-424 was downregulated; downregulation increased LPS-induced inflammation

**Table 5:** miRNA expression in sepsis-related liver dysfunction.

Author and Reference	Year of Publication	Sepsis Model	Brief Description of miRNAs in Liver during Sepsis
Yang et al. [229]	2018	In vivo	miR-155 is upregulated in septic liver; miR-155 antagomir is linked to reduced septic liver injury
Ling et al. [230]	2018	In vivo	Low miR-30a levels are linked to an increased expression of FOSL2 and the JAK/STAT pathway
Yuan et al. [231]	2019	In vivo	High miR-30a levels and diminished SOCS-1 are linked to increased hepatocyte apoptosis
Zhu et al. [86]	2019	In vivo	Low miR-98 levels are described in septic liver, cardiac and lung tissue. High miR-98 levels seem to protect from septic injury.
Zhou and Xia. [232]	2019	In vivo	High miR-103a-3p levels are reported in septic mice liver and also in the serum of septic mice and human
Chen et al. [233]	2020	In vivo	Low miR-103a-3p levels in septic live tissue compared to controls; miR-103a-3p agomiR diminished septic liver damage
Li et al. [234]	2021	In vivo	Low miR-103a-3p levels in liver and lung tissue of septic mice; diminished levels also in blood samples of human septic samples
Gu et al. [235]	2020	In vivo	miR-425-5p downregulation is linked to high liver damage in septic mice
Zhou et al. [236]	2020	In vivo	miR-10a upregulated in mimics group and downregulated in inhibitor group
Xu et al. [237]	2021	In vivo In vitro	miR-142-5p upregulation increases apoptosis and hepatocyte inflammation
Kim et al. [238]	2021	In vivo	High miR-147 levels described in liver but also in lung, kidney and stomach; miR-147 could be involved in inflammatory response
Li et al. [239]	2020	In vitro	miR-373-3p is downregulated in septic liver models; miR-373-3p reduces apoptosis of LPS THLE cells.
Li et al. [240]	2020	In vivo In vitro	miR-126-5p upregulation is linked to augmented apoptosis and low cell viability
Chen et al. [241]	2020	In vivo In vitro	miR-204-5p downregulation is associated with high LPS liver injury
Han et al. [242]	2019	In vivo	miR-9 is downregulated with high levels of MCPIP1 target, which plays a protective role in septic liver injury
Yang et al. [243]	2018	In vivo	miR-27a upregulation ameliorates liver injury
Wang et al. [244]	2020	In vivo	Inhibition of miR-640 is linked to reduction in liver damage

**Table 6:** miRNA expression in sepsis-related kidney dysfunction.

Author and Reference	Year of Publication	Sepsis Model	Brief Description of miRNAs in Renal Tissue during Sepsis
Xu, G. et al. [254]	2019	In vivo	LPS-induced inflammation stimulated the downregulation of miR-15a-5p and the upregulation of XIST and CUL3 with the inhibition of podocyte growth in animals.
Fu, D. et al. [255]	2017	In vivo	Overexpression of miRNA-21 during sepsis-induced renal cell injury may regulate PTEN/PI3K/AKT signaling and inhibit apoptosis.
Wei, W. et al. [256]	2020	In vivo	Suppression of miR-21 inhibits cell apoptosis in septic mice and LPS-stimulated human renal tubule cells. miR-21 targets the CDK6 gene inducing cellular apoptosis and leading to septic renal dysfunction
Lin Z., et al. [257]	2018	In vivo	miR-21-3p upregulated FOXO1, which enhanced cell apoptosis and the cell cycle arrest of tubule epithelial cells during sepsis-induced kidney injury.
Wei, W. et al. [258]	2021	In vitro	The expression of miR-21e5p is significantly increased in LPS-stimulated HK-2 cells. hsa_circ_0068,888 has a protective effect in LPS-induced AKI by sponging miR-21e5p.
Zou Z., et al. [259]	2020	In vivo In vitro	Upregulation of miR-21a-3p in plasma and TECs during sepsis is promoted by the internalization of plasmatic Ago2.
Zhang P. et al. [261]	2020	In vivo In vitro	Overexpression of miR-22-3p inhibits renal cell apoptosis and reduces the accumulation of cleaved caspase-3 by targeting the AIFM1 gene.
Wang X. et al. [262]	2020	In vivo In vitro	Overexpression of miR-22-3p might have a beneficial effect by attenuating sepsis- or LPS-induced inflammation and apoptosis by targeting PTEN.
Ye J. et al. [263]	2021	In vivo In vitro	Overexpression of miR-23a-3p inhibits hyperuricemia-induced renal tubular injury and renal cell apoptosis by targeting the WNT5A gene.
Chen Y. et al. [264]	2022	In vivo	The miR-26a-5p/ IL-6 axis can ameliorate sepsis-induced acute kidney injury by inhibiting renal inflammation.
Ha ZL. et al. [265]	2021	In vivo In vitro	Downregulation of miR-29b-3p causes podocyte damage by targeting the HDAC4 gene in LPS-induced AKI.
Ding G. et al. [266]	2022	In vivo In vitro	Downregulation of miR-103a-3p during sepsis is associated with the overexpression of pro-inflammatory CXCL12 gene.
Shen, Y. et al. [267]	2019	In vivo	Upregulation of miR-106a exacerbated LPS-induced the inflammation and apoptosis of TCMK-1 cells via regulating THBS2 expression
Whang, S. et al. [268]	2017	In vivo	Upregulation of miR-107 induces the secretion of TNF-α by targeting DUSP7 in endothelial cells, leading to tubule cell injury in septic AKI
Zhang H. et al. [269]	2022	In vitro	Downregulation of miR-124-3p.1 is directly related to the increased activity of LPCAT3, a key enzyme of phospholipid metabolism involved in cellular ferroptosis
Shi L. et al. [270]	2021	In vivo In vitro	Downregulation of miR-150-5p by Stat-3 activation in septic models. miR-150-5p can attenuate apoptosis in HK-2 cells, renal inflammatory response, and oxidative stress
Ren Y., et al. [272]	2017	In vivo	Downregulation of miR-155 in the kidney reduced mortality of septic mice, improved renal function, and suppressed expression of the JAK /STAT pathway
Lin Y., et al. [273]	2019	In vivo	miR-210, miR-494, and miR-205 have predictive value for the prognosis and survival of patients with sepsis-induced AKI. MiR-205 appears to be an independent risk factor for sepsis-induced AKI and its decreased expression is associated with shorter patient survival
Sang Z. et al. [273]	2020	In vivo	miR-214 could alleviate AKI in septic models by inhibiting renal autophagy through silencing PTEN expression
Guo C., et al. [275]	2022	In vivo In vitro	Downregulation of miR-214-5p dramatically reduced renal inflammation and oxidative stress, preventing septic AKI
Liu Z. et al. [276]	2020	In vivo	Overexpression of miR-452 stimulated the pro-inflammatory NF-κB pathway
Qi Y. et al. [277]	2022	In vivo	Downregulation of miR-665 was able to inhibit LPS-induced renal inflammation and apoptosis and improve renal function
Ge QM. et al. [278]	2017	In vivo	Overexpression of these miRNAs is associated with oxidative stress miR-4321 is involved in the regulation of AKT1, mTOR, and NOX5 genes miR-4270 is involved in the regulation of PPARGC1A, AKT3, NOX5, PIK3C3, and WNT1 genes

**Table 7:** miRNAs involved in sepsis-induced brain dysfunction and their target. ↑ indicates up regulation, ↓ indicates down regulation.

miRNA	Target Gene/Pathway	Upregulation/Downregulation and Its Effect on Brain Function	Author and Reference
miR-181b	-	↓miR-181b ↓Brain function	Dong et al. [19]
miR-200a-3p	NLRP3	↑miR-200a-3p ↑NLRP3 ↓Brain function	Yu et al. [21]
miR-181b	S1PR1 NCALD	↑miR-181b ↓S1PR1 and NCALD ↓Brain function	Chen et al. [20]
miR-370-3p	-	↑ miR-370-3p ↓Brain function	Visitchanakun et al. [22]
miR-147	-	↓ miR-147 ↓Brain function	Kim et al. [23]
miR-126	-	↓miR-126 - ↓Brain function	Nong et al. [24]
miR-190a-3p, miR-3085-3p	-	↑ miR-190a-3p, ↓miR-383-5p ↑ Brain function	Rani et al. [25]
miR-146a	-	↓ miR-146 a ↑ Brain function	Zou et al. [26]

**Table 8:** miRNAs involved in sepsis-induced heart dysfunction and their targets. ↑ indicates up regulation, ↓ indicates down regulation.

miRNA	Target Gene/Pathway	Upregulation/Downregulation and Its Effect on Heart Function	Author and Reference
miR-223	STAT3 Sema3A	↓miR-223 ↑STAT3 and Sema3A ↓Cardiac function	Wang et al. [54]
miR-223	FOXO1	↑miR-223 ↓FOXO1 ↑Cardiac function	Liu et al. [56]
miR-223	NF-κB	↑miR-223 ↑NF-κB -	M’baya-Moutoula et al. [57]
miR-27a	Nrf2	↑miR-27a ↑Nrf2 ↓Cardiac function	Xue et al. [58]
miR-146a	ICAM1 VCAM1	↑miR-146a ↓ICAM1 and VCAM1 ↑Cardiac function	Gao et al. [60]
miR-146a	TRAF6 IRAK1	↑miR-146a ↓ TRAF6 and IRAK1 ↑Cardiac function	An et al. [61]
miR-146a	TRAF6 IRAK1	↑miR-146a ↓TRAF6 and IRAK1 ↑Cardiac function	Xie et al. [62]
miR-146b	Notch1	↑miR-146b ↓Notch1 ↑Cardiac function	Wang et al. [63]
miR-125b	TRAF6	↓miR-125b ↑TRAF6 ↑Cardiac function	Ma et al. [64]
miR-150-5p	-	↓miR-150-5p - ↓Cardiac function	Wei et al. [65]
miR-150-5p	Akt2	↓miR-150-5p ↑Akt2 ↓Cardiac function	Zhu et al. [66]
miR-21-3p	SORBS2	↑miR-21-3p ↓SORBS2 ↓Cardiac function	Wang et al. [67]
miR-155	Pea15a	↓miR-155 ↑Pea15a ↑Cardiac function	Wang et al. [68]
miR-155	Arrb2	↑miR-155 ↓Arrb2 ↑Cardiac function	Zhou et al. [71]
miR-124a	STX2	↑miR-124 ↓STX2 ↑Cardiac function	Diao et al.. [69]
miR-135a	-	↑miR-135a - ↓Cardiac function	Zheng et al. [70]
miR-214	PTEN	↑miR-214 ↑PTEN ↑Cardiac function	Ge et al. [72]
miR-214-3p	PTEN	↑miR-214-3p ↓PTEN ↑Cardiac function	Sang et al. [73]
miR-874	AQP1	↑miR-874 ↓AQP1 ↓Cardiac function	Fang et al. [74]
miR-93-3p	TLR4	↑miR-93-3p ↓TLR4 ↑Cardiac function	Tang et al. [78]
miR-25	PTEN	↑miR-25 ↓PTEN ↑Cardiac function	Yao et al. [79]
miR-494-3p	PTEN	↑miR-494-3p ↓PTEN ↑Cardiac function	Wu et al. [80]
miR-23b	TGIF1 PTEN	↑miR-23b ↓TGIF1 and PTEN ↑Cardiac function	Zhang et al. [81]
miR-23b	TRAF6 IKKβ	↑miR-23b ↓TRAF6 and IKKβ ↑Cardiac function	Cao et al. [84]
miR-495	-	↑miR-495- ↑Cardiac function	Guo et al. [85]
miR-98	HMGA2	↑miR-98 ↓HMGA2 ↑Cardiac function	Zhu et al. [86]
miR-208-5p	SOCS2	↑miR-208-5p ↓SOCS2 ↓Cardiac function	Ouyang et al. [87]
miR-328	-	↑miR-328 - ↓Cardiac function	Sun et al. [88]
miR-29a	SIRT1	↑miR-29a ↑Cardiac function	Zhu et al. [91]
miR-29a	-	↑miR-29a ↑Cardiac function	Song et al. [92]
miR-24	XIAP	↑miR-24 ↓XIAP ↓Cardiac function	Chen et al. [93]
miR-192-5p	XIAP	↓miR-192-5p ↑XIAP ↑Cardiac function	Sun et al. [94]
miR-144-3p	-	↑miR-144-3p ↑Cardiac function	Wei et al. [95]
miR-330-5p	TRAF6	↑miR-330-5p ↓TRAF6 ↑Cardiac function	Xing et al. [96]
miR-1-5p	HSPA4	↓miR-1-5p ↑HSPA4 ↑Cardiac function	Han et al. [97]
miR-29b-3p	FOXO3A	↑miR-29b-3p ↓FOXO3A ↑Cardiac function	Li et al. [98]
miR-101-3p	DUSP1	↓miR-101-3p ↑DUSP1 ↑Cardiac function	Xin et al. [99]
miR-106b-5p	PTENP1	↑miR-106b-5p ↓PTENP1 ↑Cardiac function	Liu et al. [100]
miR-29c-3p	-	↑miR-29c-3p ↓Cardiac function	Zhang et al. [101]
miR-499a-5p	EIF4E	↑miR-499a-5p ↓EIF4E ↑Cardiac function	Yang et al. [102]

**Table 9:** miRNAs involved in sepsis-induced inflammation and their targets in blood. ↑ indicates up regulation, ↓ indicates down regulation.

miRNA	Target Gene/Pathway	Upregulation/Downregulation and Its Effect on Inflammation Function	Author and Reference
miR-494-3p	TLR6	↓miR-494-3p ↓NF-κB ↑Inflammation	Wang et al. [113]
miR-218	VOPP1	↓miR-218 ↓VOPP1 ↑Inflammation	Li et al. [114]
miR-218	RUNX2	↓miR-218 ↑RUNX2 ↑Inflammation	Zhou et al. [115]
miR-122	-	↑miR-122 ↑Inflammation	Abou El-Khier NT et al. [116]
miR-328	-	↑miR-328 ↑Inflammation	Sun et al. [117]
miR-21	-	↓miR-21 ↑Inflammation	Na et al. [118]
miR-126	EGFL7	↑miR-126 ↑Inflammation	Lin et al. [119]
miR-125a	-	↑miR-125a ↑Inflammation	Li et al. [120]
miR-146a	-	↓miR-146a ↑Inflammation	Song et al. [121]
miR-146a	NF-κB	↑miR-146a ↓NF-κB ↓Inflammation	Chen et al. [122]
miR-146a	Notch1	↑miR-146a ↓Notch1 ↓Inflammation	Bai et al. [123]
miR-146a	p38 MAPK	↑miR-146a ↓p38 MAPK ↓Inflammation	Brudecki et al. [124]
miR-146a	IRAK1 TRAF6	↑miR-146a ↓IRAK1/TRAF6 ↓Inflammation	Banarjee et al. [125]
miR-146a	STAT4	↓miR-146a ↑PRKCε/STAT4 ↑Inflammation	Mohnle et al. [126]
miR-146a	-	↑miR-146a ↓Inflammation	Paik et al. [127]
miR-146b	p38 MAPK	↑miR-146b ↓p38 MAPK ↓Inflammation	Chen et al. [122]
miR-146b	-	↑miR-146b ↑Inflammation	Zhang et al. [128]
miR-146b	NF-κB	↓miR-146b ↑NF-κB ↑Inflammation	Gao et al. [129]
miR-126	CASP3	↑miR-126 ↑CASP3 ↓Mortality	Zou et al. [130]
miR-223	HIF-1α	↑miR-223 ↓HIF-1α ↓Inflammation	Dang et al. [131]
miR-25	NOX4	↓miR-25 ↑NOX4 ↑Inflammation	Yao et al. [132]
miR-21	NLRP3 NF-κB TNFAIP3	↑miR-21 ↑NLRP3 ↑NF-κB ↓TNFAIP3 ↑Inflammation	Xue et al. [133]
miR-223	FOXO1	↑miR-223 ↓FOXO1 ↑Inflammation	Liu et al. [56]
miR-223	-	↑miR-223 ↑Inflammation	Wu et al. [135]
miR-205	HMGB1	↑miR-205 ↓HMGB1 ↓Inflammation	Zhou et al. [136]
miR-145a	Fli-1/ NF-κB	↑miR-145a ↓Fli-1/ NF-κB ↓Inflammation	Wu et al. [137]
miR-145a	TGF-β2	↓miR-145a ↑TGF-β2 ↑Inflammation	Ma et al. [138]
miR-145a	TGF-β2	↓miR-145a ↑TGF-β2 ↑Inflammation	Cao et al. [139]
miR-145a	-	↑JMJD3 ↓miR-145a ↑Inflammation	Pan et al. [140]
miR-125b	-	↑miR-125b ↑Inflammation	Zhao et al. [120]
miR-125b	PTEN/MyD88	↑miR-125b ↑PTEN ↓MyD88 ↓Inflammation	Sisti et al. [141]
miR-125b	-	↑miR-125b ↑Inflammation	Zhu et al. [142]
miR-27b	NF-κB	↑miR-27b ↓NF-κB ↓Inflammation	Sun et al. [143]
miR-203b	PTEN/MyD88	↑miR-203b ↑PTEN ↓MyD88 ↓Inflammation	Sisti et al. [141]
miR-10a	MAP3K7/ NF-κB	↓miR-10a ↑MAP3K7/ NF-κB ↑Inflammation	Zheng et al. [144]
miR-155	-	↑miR-155 ↑Inflammation	Liu et al. [145]
miR-7110-5p	-	↑miR-7110-5p ↑Inflammation	Zhang et al. [146]
miR-19a	-	↑miR-19a ↑Inflammation	Jiang et al. [147]
miR-19b-3p	-	↑miR-19b-3p ↓Inflammation	Xu et al. [148]
miR-150	ARG1	↑miR-150 ↓ARG1 ↓Inflammation	Liu et al. [149]
miR-375	JAK2-STAT3	↑miR-375 ↓JAK2-STAT3 ↓Inflammation	Sheng et al. [150]
miR-21 miR-181b	NFI-A	↓miR-21 and miR-181b ↓NFI-A ↓Inflammation	McClure et al. [151]
miR-21 miR-181b	STAT3 and C/EBPβ	↓miR-21 and miR-181b ↓STAT3 and C/EBPβ ↓Inflammation	McClure et al. [152]
miR-143	-	↑miR-143 ↑Inflammation	Han et al. [153]
miR-20b-5p	-	↑circDNMT3B ↓miR-20b-5p ↓Inflammation	Liu et al. [154]
miR-17-5p	TLR4	↑miR-17-5p ↓TLR4 ↓Inflammation	Ji et al. [155]
miR-26b	SELP	↓miR-26b ↑SELP ↑Inflammation	Szilagyi et al. [156]
miR-96-5p	NAMPT	↑miR-96-5p ↓NAMPT ↓Inflammation	Chen et al. [157]
miR-27a	PPAR γ	↓miR-27a ↑PPAR γ ↓Inflammation	Wang et al. [158]
miR-27a	TAB3	↓miR-27a ↑TAB3 ↓Inflammation	Yang et al. [159]
miR-574-5p	-	↑miR-574-5p ↑Inflammation	Wang et al. [43]
miR-181b	Importin-α3 NFkβ	↑miR-181b ↓Importin-α3 and NFkβ ↓Inflammation function	Sun et al. [45]
miR-346	SMAD3	↑miR-346 ↓SMAD3 ↑Inflammation	Yang et al. [160]
miR-148a-3p	Notch1	↑miR-148a-3p ↑Notch1 ↑Inflammation	Bai et al. [161]
miR-218-5p	HO-1	↓miR-218-5p ↑HO-1 ↓Inflammation	Zhang et al. [162]
miR-1298-5p	SOCS6	↓miR-1298-5p ↓STAT3 ↓inflammation	Ma et al. [163]
miR-608	ELANE	↑miR-608 ↓Inflammation	Gu et al. [164]
miR-124	-	↓miR-124 ↑Inflammation	Zhang et al. [165]
miR-29a	-	↑miR-29a ↑Inflammation	Huo et al. [166]
miR-155	NF-κB	↑miR-155 ↓NF-κB ↑Inflammation	Cao et al. [167]
miR-155	-	↑miR-155 ↑Inflammation	Ma et al. [168]
miR-30a	MD-2	↑miR-30a ↓MD-2 ↑Inflammation	Wang et al. [169]
miR-339-5p	HMGB1	↑miR-339-5p ↓HMGB1 ↓Inflammation	Mei et al. [170]
miR-99b	MFG-E8	↑miR-99b ↓MFG-E8 ↑Inflammation	Wang et al. [171]
miR-215-5p	ILF3 and LRRFIP1	↓miR-215-5p ↑ILF3 and LRRFIP1 ↑Inflammation	Yao et al. [172]
miR-15a	-	↑miR-15a ↑Inflammation	Wang et al. [173]
miR-206	-	↑miR-206 ↑Inflammation	Liang et al. [174]
miR-92a-3p	LCN2	↓miR-92a-3p ↓LCN2 ↓Inflammation	Wang et al. [175]

**Table 10:** miRNAs involved in sepsis-induced lung dysfunction and their targets. ↑ indicates up regulation, ↓ indicates down regulation.

miRNA	Target Gene/Pathway	Upregulation/Downregulation and Its Effect on Lung Function	Author and Reference
miR-155	TAB2	↑miR-155 ↓TAB2 ↑Pulmonary function	Liu et al. [191]
miR-155	SHIP1 SOCS1	↑miR-155 ↓SHIP1 and SOCS1 ↓Pulmonary function	Jiang et al. [192]
miR-155	TREM1	↑miR-155 ↑TREM1 ↓Pulmonary function	Yuan et al. [193]
miR-155	IRF2BP2	↑miR-155 ↓IRF2BP2 ↓Pulmonary function	Li et al. [195]
miR-155	-	↓miR-155 - -	Han et al. [196]
miR-146	Akt2	↑miR-146 ↓Akt2 ↑Pulmonary function	Vergadi et al. [197]
miR-146	IRAK1 TRAF6	↑miR-146 ↓IRAK1 and TRAF6 ↑Pulmonary function	Zeng et al. [198]
miR-144-3p	CAV2	↑miR-144-3p ↓CAV2 ↓Pulmonary function	Xu et al. [199]
miR-19a-3p	USP13	↑miR-19a-3p ↓USP13 ↓Pulmonary function	Ren et al. [200]
miR-199a	SIRT1	↓miR-199a ↑SIRT1 ↑Pulmonary function	Liu et al. [201]
miR-34a	SIRT1 ATG4B	↓miR-34a ↑SIRT1 and ATG4B ↑Pulmonary function	Chen et al. [202]
miR-34b-5p	TUG1	↓miR-34b-5p ↑TUG1 ↑Pulmonary function	Qiu et al. [221]
miR-132	SIRT1	↑miR-132 ↓SIRT1 ↓Pulmonary function	Zhu et al. [203]
miR-30d-5p	SIRT1 SOCS1	↑miR-30d-5p ↑SIRT1 and SOCS1 ↓Pulmonary function	Jiao et al. [204]
miR-1298-5p	SOCS6	↑miR-1298-5p ↓SOCS6 ↓Pulmonary function	Ma et al. [226]
miR-92a-3p	AKAP1	↑miR-92a-3p ↓AKAP1 ↓Pulmonary function	Wang et al. [205]
miR-23a	PTEN	↑miR-23a ↓PTEN ↑Pulmonary function	Yang et al. [206]
miR-218	RUNX2	↑miR-218 ↓RUNX2 ↑Pulmonary function	Zhou et al. [227]
miR-539-5p	ROCK1	↑miR-539-5p ↓ROCK1 ↑Pulmonary function	Meng et al. [207]
miR-145	TGF-β2	↓miR-145 ↑TGF-β2 ↓Pulmonary function	Cao et al. [139]
miR-124	MAPK14	↓miR-124 ↑MAPK14 ↓Pulmonary function	Pan et al. [208]
miR-483-5p	PIAS1	↑miR-483-5p ↓PIAS1 ↓Pulmonary function	Leng et al. [209]
miR-497-5p	IL2RB	↓miR-497-5p ↑IL2RB ↑Pulmonary function	Lou et al. [210]
miR-802	Peli2	↑miR-802 ↓Peli2 ↑Pulmonary function	You et al. [211]
miR-326	TLR4	↑miR-326 ↓TLR4 ↑Pulmonary function	Wang et al. [212]
miR-129-5p	HMGB1	↑miR-129-5p ↓HMGB1 ↑Pulmonary function	Yang et al. [213]
miR-129	TAK1	↑miR-129 ↓TAK1 ↑Pulmonary function	Yao et al. [215]
miR-490	MRP4	↑miR-490 ↓MRP4 ↑Pulmonary function	Lin et al. [214]
miR-127	CD64	↑miR-127 ↓CD64 ↑Pulmonary function	Xie et al. [216]
miR-125-5p	TOP2A	↑miR-125-5p ↓TOP2A ↑Pulmonary function	Jiang et al. [217]
miR-499-5p	SOX6	↑miR-499-5p ↓SOX6 ↑Pulmonary function	Zhang et al. [218]
miR-942-5p	TRIM37	↑miR-942-5p ↓TRIM37 ↑Pulmonary function	Lu et al. [219]
miR-16-5p	BRD4	↑miR-16-5p ↓BRD4 ↑Pulmonary function	Yin et al. [220]
miR-195-5p	PDK4	↑miR-195-5p ↓PDK4 ↓Pulmonary function	Wang et al. [222]
miR-152-3p	PDK4	↓miR-152-3p ↑PDK4 ↑Pulmonary function	Zhu et al. [223]
miR-128-3p	SIRT1	↑miR-128-3p ↓SIRT1 ↓Pulmonary function	Xie et al. [224]
miR-424	ROCK2	↓miR-424 ↑ROCK2 ↓Pulmonary function	Chen et al. [225]

**Table 11:** miRNAs involved in sepsis-induced liver dysfunction and their targets. ↑ indicates up regulation, ↓ indicates down regulation.

miRNA	Target Gene/Pathway	Upregulation/Downregulation and Its Effect on Liver Function	Author and Reference
miR-155	Nrf-2	↑miR-155 ↓Nrf-2 ↓Liver function	Yang et al. [229]
miR-30a	FOSL2	↓miR-30a ↑FOSL2 ↓Liver function	Ling et al. [230]
miR-30a	SOCS-1	↑ miR-30a ↓SOCS-1 ↓Liver function	Yuan et al. [231]
miR-98	HMGA2	↓miR-98 ↑HMGA2 ↓Liver function	Zhu et al. [86]
miR-103a-3p	FBXW7	↑miR-103a-3p ↓FBXW7 ↓Liver function	Zhou and Xia. [232]
miR-103a-3p	HMGB1	↓miR-103a-3p ↓HMGB1 with miR-103a-3p agomiR ↓Liver function	Chen et al. [233]
miR-103a-3p	HMGB1	↓miR-103a-3p ↑HMGB1 ↓Liver function	Li et al. [234]
miR-425-5p	RIP1	↓miR-425-5p ↑RIP1 ↓Liver function	Gu et al. [235]
miR-10a	CYP2E1, TGF-β1, and Smad2	↑miR-10a in mimics and↓in inhibitor ↑CYP2E1, TGF-β1, and Smad2 in mimics ↓Liver function with miR-10a silencing	Zhou et al. [236]
miR-142-5p	-	↑miR-142-5p ↓Liver function	Xu et al. [237]
miR-147	-	↑miR-147 ↓Liver function	Kim et al. [238]
miR-373-3p	TRIM8	↓miR-373-3p ↑TRIM8 ↑Liver function (protective role against apoptosis)	Li et al. [239]
miR-126-5p	-	↑miR-126-5p - ↓Liver function	Li et al. [240]
miR-204-5p	TRPM7	↓miR-204-5p ↑TRPM7 ↓Liver function	Chen et al. [241]
miR-9	MCPIP1	↓miR-9 ↑MCPIP1 ↑Liver function	Han et al. [242]
miR-27a	TAB3	↑miR-27a ↓TAB3 ↑Liver function	Yang et al. [243]
miR-640	LRP1	↑miR-640 ↓LRP1 ↓Liver function	Wang et al. [244]

**Table 12:** miRNAs involved in sepsis-induced kidney dysfunction and their targets. ↑ indicates up regulation, ↓ indicates down regulation.

miRNA	Target Gene/Pathway	Upregulation/Downregulation and Its Effect on Renal Function	Author and Reference
miR-15a-5p	XIST CUL3	↓miR-15a-5p ↑XIST and CUL3 ↓Renal function	Xu, G. et al. [254]
miR-21	PTEN/PI3K/AKT	↑miR-21 ↓PTEN/PI3K/AKT ↑Renal function	Fu, D. et al. [255]
miR-21	CDK6	↓miR-21 ↓CDK6 ↑Renal function	Wei, W. et al. [256]
miR-21-3p	FOXO1	↑miR-21-3p ↑FOXO1 ↓Renal function	Lin Z., et al. [257]
miR-21e5p	-	↑hsa_circ_0068,888 ↓miR-21e5p ↑Renal function	Wei, W. et al. [258]
miR-21a-3p	-	↑Ago2 ↑miR-21a-3p ↓Renal function	Zou Z., et al. [259]
miR-22-3p	AIFM1	↑miR-22-3p ↓AIFM1 ↑Renal function	Zhang P. et al. [261]
miR-22-3p	PTEN	↑miR-22-3p ↓PTEN ↑Renal function	Wang X. et al. [262]
miR-23a-3p	WNT5A	↑miR-23a-3p ↓WNT5A ↑Renal function	Ye J. et al. [263]
miR-26a-5p	IL-6	↑miR-26a-5p ↓IL-6 ↑Renal function	Chen Y. et al. [264]
miR-29b-3p	HDAC4	↓miR-29b-3p ↑HDAC4 ↓Renal function	Ha ZL. et al. [265]
miR-103a-3p	CXCL12	↓miR-103a-3p ↑CXCL12 ↓Renal function	Ding G. et al. [266]
miR-106a	THBS2	↑miR-106a ↑THBS2 ↓Renal function	Shen, Y. et al. [267]
miR-107	DUSP7	↑miR-107 ↓DUSP7 ↓Renal function	Whang, S. et al. [268]
miR-124-3p.1	LPCAT3	↓miR-124-3p.1 ↑LPCAT3 ↓Renal function	Zhang H. et al. [269]
miR-150-5p	MEKK3/JNK	↓miR-150-5p ↑MEKK3/JNK ↓Renal function	Shi L. et al. [270]
miR-155	JAK /STAT	↓miR-155 ↓JAK /STAT ↑Renal function	Ren Y., et al. [272]
miR-210 miR-494 miR-205	- - -	↑miR-210 ↑Renal function ↑miR-494 ↑Renal function ↓miR-205 ↓Renal function	Lin Y., et al. [273]
miR-214	PTEN	↑miR-214 ↓PTEN ↑Renal function	Sang Z. et al. [273]
miR-214-5p	-	↓miR-214-5p ↑Renal function	Guo C., et al. [275]
miR-452	-	↑miR-452 ↑NF-κB pathway ↓Renal function	Liu Z. et al. [276]
miR-665	BCL-2	↑miR-665 ↓BLC-2 ↓Renal function	Qi Y. et al. [277]
miR-4321 miR-4270	AKT1, mTOR, NOX5 PPARGC1A, AKT3, NOX5, PIK3C3, WNT1	↑miR-4321 ↑AKT1, mTOR, NOX5 ↓Renal function ↑miR-21 ↑PPARGC1A, AKT3, NOX5, PIK3C3, WNT1 ↓Renal function	Ge QM. et al. [278]

## Data Availability

Not applicable.

## Abbreviations

AIFM1	Apoptosis-inducing mitochondrial factor
AKAP1	A-kinase anchor protein 1
AKT1	Serine/Threonine Kinase 1
AKT2	Serine/Threonine Kinase 2
AKT3	Serine/Threonine Kinase 3
AQP1	Aquaporin 1
ARG1	Arginase 1
ARRB2	Arrestin Beta 2
ATG4B	Autophagy-Related 4B
BRD4	Bromodomain-containing protein 4
C/EBPβ	CCAAT-enhancer-binding protein beta
CASP3	Caspase 3
CAV2	Caveolin-2;
CD64	Cluster of Differentiation 64
CDK6	Cyclin-Dependent Kinase 6
CUL3	Cullin 3;
CXCL12	C-X-C Motif Chemokine Ligand 12
CYP2E1	Cytochrome P450 2E1
DUSP1	Dual-Specificity Phosphatase 1
DUSP7	Dual-Specificity Phosphatase 7
EGFL7	EGF-Like Domain Multiple 7
EIF4E	Eukaryotic Initiation Factor 4E
ELANE	Elastase, Neutrophil-Expressed
FBXW7	F-Box And WD Repeat Domain Containing 7
Fli-1	Friend leukemia integration 1
FOSL2	FOS-Like 2
FOXO1	Forkhead Box O1
FOXO3A	Forkhead Box O3A
HDAC4	Histone Deacetylase 4
HIF-1α	Hypoxia-inducible factor 1-alpha
HMGA2	High-mobility group AT-hook 2
HMGB1	High mobility group box 1
HO-1	Heme oxygenase-1
HSPA4	Heat shock 70 kDa protein 4
ICAM1	Intercellular Adhesion Molecule 1
IKKβ	Inhibitor of nuclear factor kappa-B kinase subunit beta
IL2RB	Interleukin 2 Receptor Subunit Beta
ILF3	Interleukin Enhancer-Binding Factor 3
IRAK1	Interleukin 1 Receptor-Associated Kinase 1
IRF2BP2	Interferon regulatory factor 2-binding protein 2
JAK	Janus Kinase
JAK2	Janus Kinase 2
JNK	c-Jun N-terminal kinase
LCN2	Lipocalin-2;
LPCAT3	Lysophosphatidylcholine acyltransferase 3
LRP1	Low-density lipoprotein receptor-related protein 1
LRRFIP1	LRR Binding FLII Interacting Protein 1
MAP3K7	Mitogen-activated protein kinase kinase kinase 7
MAPK14	Mitogen-Activated Protein Kinase 14
MCPIP1	Monocyte Chemotactic Protein-Induced Protein 1
MD-2	Myeloid Differentiation factor 2
MFG-E8	Milk fat globule-EGF factor 8
MRP4	Multidrug resistance protein 4
mTOR	Mammalian Target Of Rapamycin
MyD88	Myeloid differentiation primary response 88
NAMPT	Nicotinamide Phosphoribosyltransferase
NCALD	Neurocalcin Delta
NFI-A	Nuclear Factor I A
NF-κB	Nuclear Factor kappa B
NLRP3	NLR family pyrin domain containing 3
Notch1	Neurogenic locus notch homolog protein 1
NOX4	NADPH oxidase 4
NOX5	NADPH oxidase 5
Nrf2	Nuclear factor erythroid 2-related factor 2
p38 MAPK	p38 mitogen-activated protein kinase
PDK4	Pyruvate dehydrogenase lipoamide kinase isozyme 4
Pea15a	Proliferation and apoptosis adaptor protein 15A
Peli2	Pellino E3 Ubiquitin Protein Ligase Family Member 2
PI3K	Phosphoinositide 3-kinases
PIAS1	Protein Inhibitor Of Activated STAT 1
PIK3C3	Phosphatidylinositol 3-Kinase Catalytic Subunit Type 3
PPAR γ	Peroxisome proliferator-activated receptor gamma
PPARGC1A	PPARG Coactivator 1 Alpha
PTEN	Phosphatase and tensin homolog
PTENP1	Hosphatase and tensin homolog pseudogene 1
RIP1	Receptor-interacting serine/threonine-protein kinase 1
ROCK1	Rho-associated kinase 1
ROCK2	Rho-associated kinase 2
RUNX2	Runt-related transcription factor 2
S1PR1	Sphingosine-1-phosphate receptor 1
SELP	Selectin P
SEMA3A	Semaphorin 3A
SHIP1	Src homology 2 domain containing inositol polyphosphate 5-phosphatase 1
SIRT1	Sirtuin 1
SMAD2	Small Mother Against Decapentaplegic 2
SMAD3	Small Mother Against Decapentaplegic 3
SOCS1	Suppressor of cytokine signaling 1
SOCS2	Suppressor of cytokine signaling 2
SOCS6	Suppressor of cytokine signaling 6
SORBS2	Sorbin and SH3 Domain Containing 2
SOX6	SRY-Box Transcription Factor 6
STAT3	Signal transducer and activator of transcription 3
STAT4	Signal transducer and activator of transcription 4
STX2	Syntaxin 2
TAB2	TGF-beta activated kinase 1 (MAP3K7)-binding protein 2
TAB3	TGF-beta activated kinase 1 (MAP3K7)-binding protein 3
TAK1 [MAP3K7]	Mitogen-activated protein kinase kinase kinase 7
TGF-β1	Transforming Growth Factor Beta Receptor 1
TGF-β2	Transforming Growth Factor Beta Receptor 2
TGIF1	TGFB-Induced Factor Homeobox 1
THBS2	Thrombospondin 2
TLR4	Toll-Like Receptor 4
TLR6	Toll-Like Receptor 6
TNFAIP3	TNF alpha-induced protein 3
TOP2A	DNA Topoisomerase II Alpha
TRAF6	TNF Receptor Associated Factor 6
TREM1	Triggering Receptor Expressed On Myeloid Cells 1
TRIM37	Tripartite Motif Containing 37
TRIM8	Tripartite Motif Containing 8
TRPM7	Transient Receptor Potential Cation Channel Subfamily M Member 7
TUG1	Taurine Upregulated 1
USP13	Ubiquitin Specific Peptidase 13
VCAM1	Vascular Cell Adhesion Molecule 1
VOPP1	VOPP1 WW Domain-Binding Protein
WNT1	Wnt family member 1
WNT5A	Wnt Family Member 5A
XIAP	X-Linked Inhibitor Of Apoptosis
XIST	X-inactive-specific transcript
